# Comparing the Efficacy of Renal Artery Denervation in Uncontrolled Hypertension: A Systematic Review and Network Meta-Analysis

**DOI:** 10.7759/cureus.70805

**Published:** 2024-10-04

**Authors:** Alaa Abdrabou Abouelmagd, Maged Elsayed Hassanein, Rana Ibrahim Abdalla Shehata, Omar A Kaoud, Heba Hamouda, Omar F Abbas, Mohab Gaballah

**Affiliations:** 1 Medicine, South Valley University, Qena, EGY; 2 Cardiology, Medical Research Group of Egypt, Negida Academy, Arlington, USA; 3 Medicine, Zagazig University, Zagazig, EGY; 4 Nephrology, Ain Shams University, Cairo, EGY; 5 Medicine, Tanta University, Tanta, EGY; 6 Medicine, Menoufiya University, Shibin Al Kawm, EGY; 7 Medicine, Al-Azhar University, Cairo, EGY; 8 General Surgery, Ain Shams University, Cairo, EGY

**Keywords:** hypertension, network meta-analysis, radiofrequency, renal artery, renal denervation

## Abstract

The study aims to compare the outcomes of different renal denervation (RDN) procedures in the treatment of uncontrolled hypertension. We searched Scopus, PubMed, Web of Science, and Cochrane for RCTs evaluating different procedures of RDN for hypertension. The outcomes of this study were systolic blood pressure (SBP) daytime, diastolic blood pressure (DBP) daytime, SBP nighttime, DBP nighttime, SBP 24-hour, DBP 24-hour, SBP home, DBP home, SBP office, and DBP office. We did a frequentist network meta-analysis of 38 published RCTs evaluating the efficacy of different renal artery denervation procedures for uncontrolled hypertension compared to sham procedures or standardized stepped-care antihypertensive treatment (SSAHT). Radiofrequency (RF) alone showed a statistically significant reduction in DBP (24 hours), DBP (daytime), and DBP (nighttime): standardized mean difference (SMD): -2.01 (95% CI: (-3.34; -0.68)), SMD: -4.36 (95% CI: (-8.28; -0.44)), and SMD: -3.50 (95% CI: (-6.23; -0.76)), respectively, and showed a statistically significant reduction in SBP (24 hours), SBP (daytime), and SBP (nighttime): SMD: -3.93 (95% CI: (-6.01; -1.84)), SMD: -5.88 (95% CI: (-9.91; -1.85)), and SMD: -5.79 (95% CI: (-10.0; -1.58)), respectively. RF added to SSAHT has statistical significance in the reduction of DBP (nighttime), SBP (daytime), SBP (home), and SBP (nighttime) with a SMD of -7.63 (95% CI: (-14.21; -1.06)), SMD of -10.56 (95% CI: (-21.03; -0.08)), SMD of -23.20 (95% CI: (-36.72; -9.26)), and SMD of -14.03 (95% CI: (-25.43; -2.63)), respectively. We found that renal denervation, especially by RF, when added to SSAHT may be a promising therapeutic option for patients with treatment-resistant hypertension, particularly in cases where medication alone fails to achieve adequate blood pressure control.

## Introduction and background

Hypertension is what we call the “silent killer.” Its prevalence is increasing worldwide due to the aging population and obesity [1]. There are more than 65 million individuals in the United States (US) [2] and around a billion individuals worldwide suffering from hypertension [3]. Resistant hypertension (RH) is defined as blood pressure (BP) higher than 140/90 mm Hg (or greater than 130/80 mm Hg in patients with diabetes or chronic kidney disease (CKD) [4] despite using three or more antihypertensive medications from at least three different classes, including a diuretic [5]. Risk factors contributing to RH are diabetes mellitus, CKD, and sleep apnea. There is a relationship between RH and cardiovascular complications, as BP is strongly and directly related to vascular mortality [6]. Ten percent of the patients treated for high BP in primary care practices suffer RH and 40% in nephrology specialty clinics [1,7]. Controlling BP is essential because patients with uncontrolled hypertension have a higher risk of cerebrovascular and cardiovascular complications and mortality than normotensive individuals, whereas mortality risk in patients with controlled hypertension is the same as in normotensive individuals [8]. In patients for whom the pharmacological treatment is limited or ineffective, the sympathetic nervous system plays a role in controlling RH, particularly the sympathetic connection between the kidneys and the brain [9].

Historically, surgical sympathectomy was done as a management for RH and led to a significant reduction in BP but with high surgical morbidity [10-12]. Percutaneous renal sympathectomy has come up as a safer way, although an invasive procedure using radiofrequency (RF) probes to ablate the sympathetic fibers along the renal artery [5] followed by catheter-based RF denervation of the renal arteries as a potential treatment for RH, and is already being used in over 80 countries, including parts of Europe, South America, Australia, and Canada [13-15]. Proof-of-concept trials reported a dramatic lowering effect in BP in patients treated with RF catheter-based renal denervation (RDN) [16,17]. Nonetheless, the unsatisfactory results of the randomized, sham-controlled SYMPLICITY HTN-3 study dampened expectations [18]. However, it was shown that a variety of confounding factors could explain these results [19]. On the other hand, proof-of-concept trials involving second-generation RF and ultrasound-based RDN devices have shown promising results [20,21] and adequately powered trials [22-25]. In this study, we aim to assess the efficacy of different RDN procedures in the treatment of uncontrolled hypertension.

## Review

Methods

In this systematic review and network meta-analysis, we followed the 2020 updated version of the Preferred Reporting Items for Systematic Reviews and Meta‐analyses (PRISMA) [26]. We registered the protocol on PROSPERO with a registration number of CRD42023457751.

Eligibility Criteria

We included all studies that satisfied the following criteria. The population consisted of hypertensive patients. The intervention involved various renal artery denervation procedures. The studies compared these procedures to either a sham procedure or standardized stepped-care antihypertensive treatment (SSAHT). Studies were eligible if they reported at least one of the following outcomes: SBP or DBP during daytime, nighttime, over 24 hours, at home, or in the office. Furthermore, only randomized controlled trials (RCTs) were included.

We excluded studies that were not in the English language and studies that used observational design.

Literature Search

PubMed, Scopus, Web of Science, and Cochrane Library from inception to September 2023 were searched for English language articles using terms related to ((renal AND denervation) AND ((resistant OR uncontrolled) AND hypertension) AND (("randomized controlled trial" OR (random AND allocation))). Four researchers independently screened the titles and abstracts of all identified studies. Full-text articles of potentially eligible studies were sorted out and assessed for inclusion. Conflicts were resolved by discussion.

Data Extraction

Data from the included studies was extracted by two reviewers using a standardized data extraction form. A third reviewer verified the extracted information. Conflicts were resolved by consensus. The extracted data from each of the RCTs included the following: (1) study design, (2) baseline characteristics of patients, (3) risk of bias domains, and (4) study outcomes. Data are extracted in mean and standard deviation (SD) and if any study reports other forms such as median, CI, or IQR they were treated to mean and SD by the conversion tools. All the data would be extracted at the last point in the study.

Outcome Measures

Our efficacy outcomes were the change from baseline in DBP 24 hours, DBP daytime, DBP home, DBP nighttime, DBP office, systolic blood pressure (SBP) 24 hours, SBP daytime, SBP home, SBP nighttime, and SBP office.

Synthesis of Results

In our research, we carefully analyze the efficacy of different RDN procedures in the treatment of hypertension patients. We did both types of analysis (pairwise and frequentist). We used a random-effect model for all the outcomes. We expressed outcome measures as mean differences. We did Egger tests to check if there was any publication bias in the studies. All of these analyses were conducted using R (version 4.4.1, R Core Team, 2024, R Foundation for Statistical Computing, Vienna, Austria), RStudio (version 2024.01.0+351, RStudio, 2024, Boston, USA), and the netmeta package (version 2.8, Schwarzer, 2024, Germany).

Heterogeneity Assessment

To evaluate statistical heterogeneity among studies, the chi-square test (Cochrane Q test) was used. The chi-square statistic, Cochrane Q, was then used to calculate the I-squared (I^2^) according to the equation: \[I^2 = \left( \frac{Q - df}{Q} \right) \times 100\%\]A chi-square p-value less than 0.1 was considered significant heterogeneity. I-square values ≥50% were indicative of substantial heterogeneity.

Risk of Bias Across Studies

We used the Cochrane Risk of Bias 2 (ROB 2) tool to assess the quality of the included RCTs [27]. There are five key domains (bias arising from the randomization process, deviations from intended interventions, missing outcome data, selection of reported results, and measurement of the outcome). Each domain is assessed using questions and overall evaluation of "low risk," "some concerns," or "high risk" of bias.

Results

Literature Search Results and Study Selection

**Figure 1:**
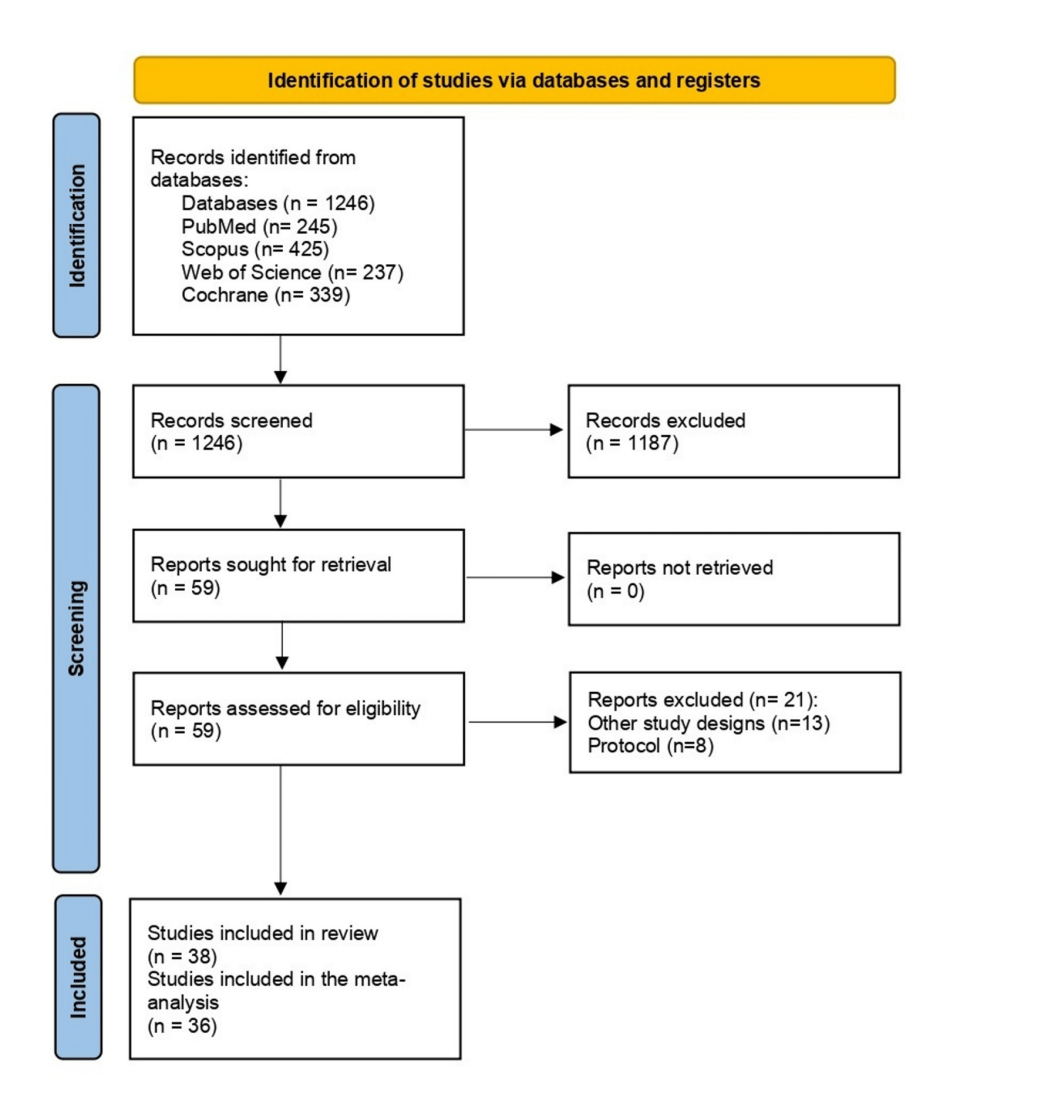
PRISMA flow diagram PRISMA: Preferred Reporting Items for Systematic Reviews and Meta‐analyses

Our literature search process retrieved 1246 records. Following titles and abstract screening, 59 articles were eligible for full-text screening. From these 59 studies, 38 RCTs were included in this review and 36 in the meta-analysis. We excluded 21 studies because they did not meet the inclusion criteria. Also, the references of the included studies were manually searched, and no further articles were included. The flow chart of the study selection process is shown in the PRISMA flow diagram in Figure 1.

Study Characteristics

The 38 RCTs included 4498 patients with hypertension. Eligible patients in the included studies were aged 18 to 75 years with a mean age of 55 years. The characteristics of the included studies and the efficacy points, the baseline of mean diastolic blood pressure (DBP) per 24 hours, and SBP per 24 hours are summarized in supplemental material 1.

Risk of Bias Within Studies

The quality of included studies ranged from low to high quality according to the Cochrane Risk of Bias Assessment tool 2. Fourteen studies were of high risk. Seventeen studies were of low risk, and seven studies had some concerns The detailed risk of bias domains by study ID is reported in supplemental material 2, and the risk of bias summary is in supplemental material 3.

Efficacy Outcome Measures

**Figure 2:**
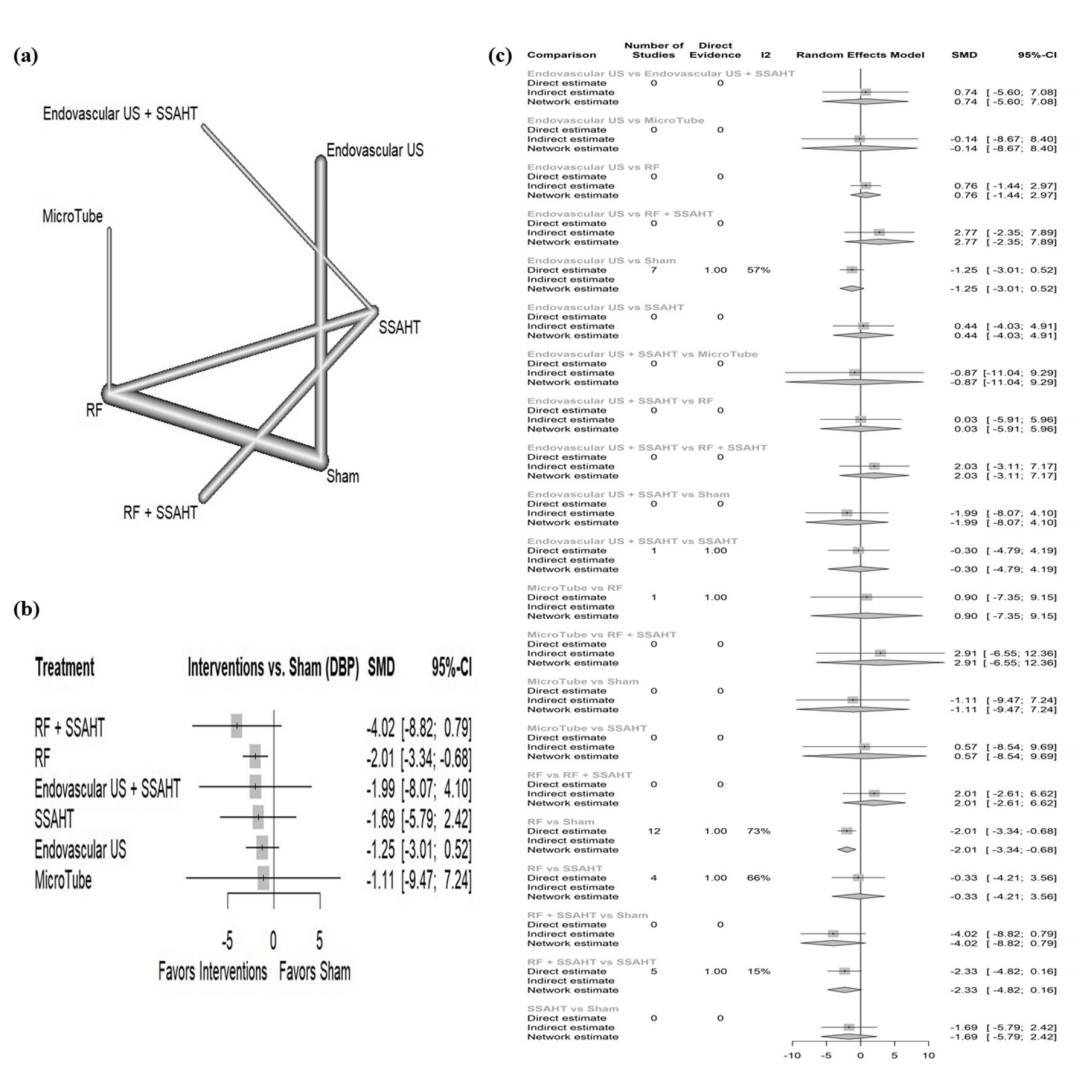
(a) Network geometry of DBP (24 hours). (b) Forest plot of change of DBP (24 hours) by different renal denervation interventions. (c) Direct versus indirect evidence of the change in DBP (24 hours) SSAHT: standardized stepped-care antihypertensive treatment; RF: radiofrequency; DBP: diastolic blood pressure; SMD: standardized mean difference

DBP per 24 hours: The network plot (Figure 2a) displays the direct comparisons between the different interventions included in the analysis. Figure 2b shows the results comparing various interventions against the sham procedure to affect DBP per 24 hours. RF + SSAHT showed the largest reduction in DBP per 24 hours with a standardized mean difference (SMD) of -4.02 (95% CI: (-8.82; 0.79)) with no statistical significance. The intervention RF alone demonstrated a statistically significant reduction in DBP per 24 hours (SMD: -2.01, 95% CI: (-3.34; -0.68)). Other interventions show no statistical significance. We assessed the direct evidence proportion for each network estimate using a random-effect model. Figure 2c presents the percentage of direct and indirect evidence contributions for each comparison. Publication bias was assessed using a comparison-adjusted funnel plot (supplemental material 4); the funnel plot shows asymmetry (p = 0.088).

**Figure 3:**
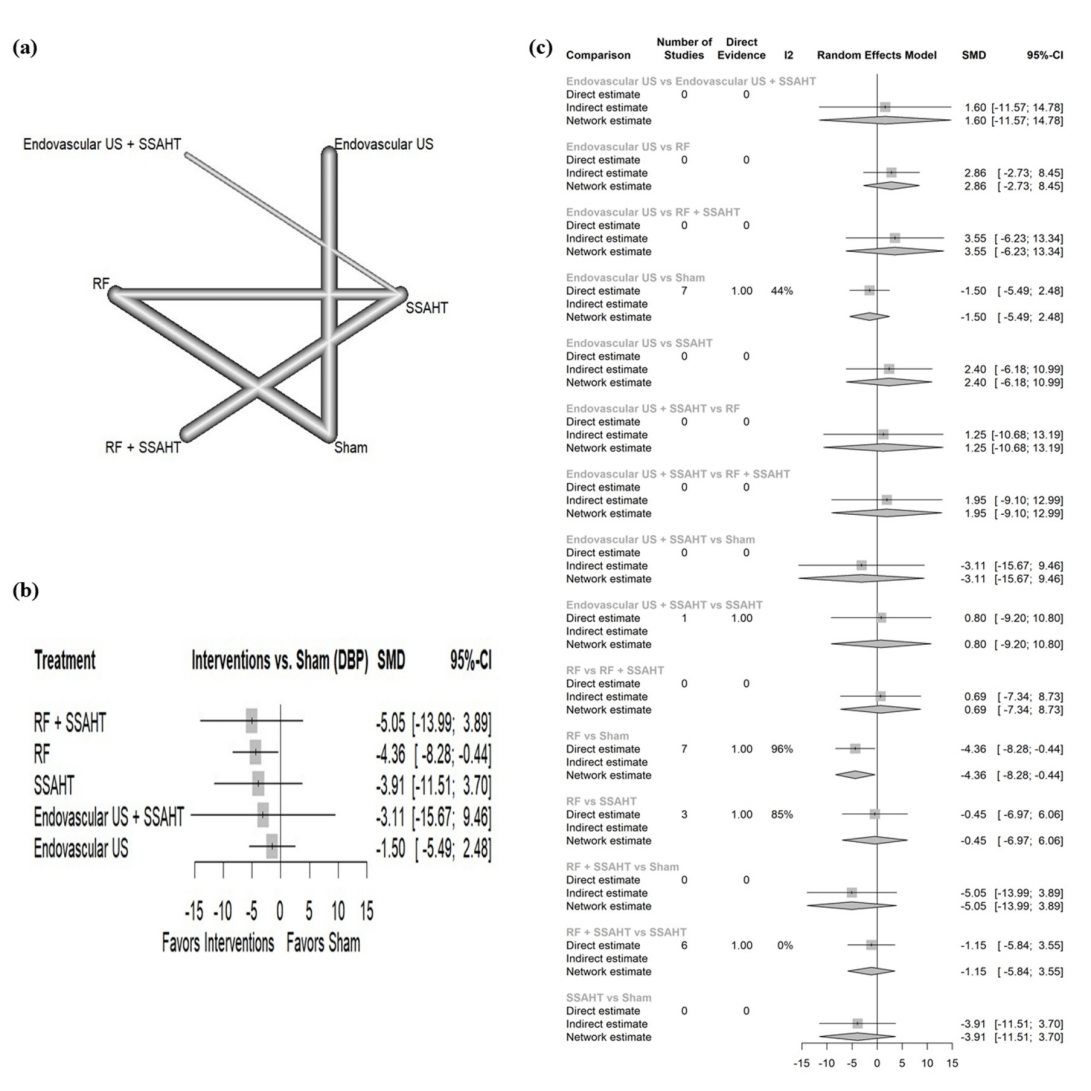
(a) Network geometry of DBP (daytime). (b) Forest plot of change of DBP (daytime) by different renal denervation interventions. (c) Direct versus indirect evidence of the change in DBP (daytime) SSAHT: standardized stepped-care antihypertensive treatment; RF: radiofrequency; DBP: diastolic blood pressure; SMD: standardized mean difference

DBP daytime: The network plot (Figure 3a) displays the direct comparisons between the different interventions included in the analysis. Figure 3b shows the results comparing various interventions against the sham procedure to affect DBP during the daytime. RF + SSAHT showed the largest reduction in DBP (daytime) with an SMD of -5.05 (95% CI: (-13.99; 3.89)) with no statistical significance. The intervention RF alone demonstrated a statistically significant reduction in DBP (daytime) (SMD: -4.36, 95% CI: (-8.28; -0.44)). Other interventions show no statistical significance. Figure 3c presents the percentage of direct and indirect evidence contributions for each comparison. Publication bias was assessed using a comparison-adjusted funnel plot (supplemental material 5); the funnel plot shows significant asymmetry (p = 0.0294).

**Figure 4:**
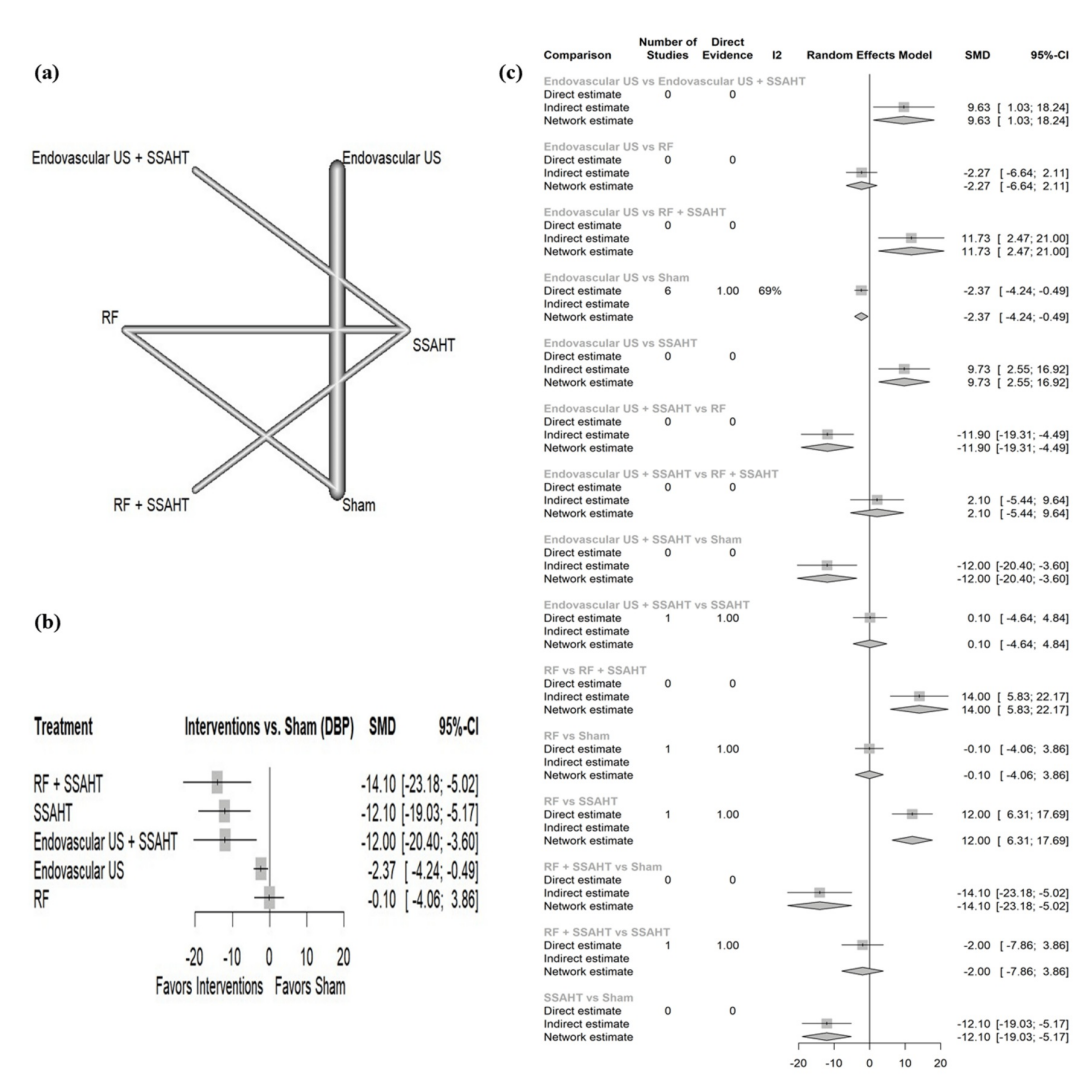
(a) Network geometry of DBP (home). (b) Forest plot of change of DBP (home) by different renal denervation interventions. (c) Direct versus indirect evidence of the change in DBP (home) SSAHT: standardized stepped-care antihypertensive treatment; RF: radiofrequency; DBP: diastolic blood pressure; SMD: standardized mean difference

DBP home: The network plot (Figure 4a) displays the direct comparisons between the different interventions included in the analysis. Figure 4b shows the results comparing various interventions against the sham procedure to affect DBP at home. RF + SSAHT showed the largest reduction in DBP (home) with an SMD of -14.10 (95% CI: (-23.18; -5.02)). All interventions have statistical significance except RF alone, which has no statistically significant reduction in DBP (home) (SMD: -0.10, 95% CI: (-4.06; 3.86)). Figure 4c presents the percentage of direct and indirect evidence contributions for each comparison. Publication bias was assessed using a comparison-adjusted funnel plot (supplemental material 6); the funnel plot shows symmetry (p = 0.1634).

**Figure 5:**
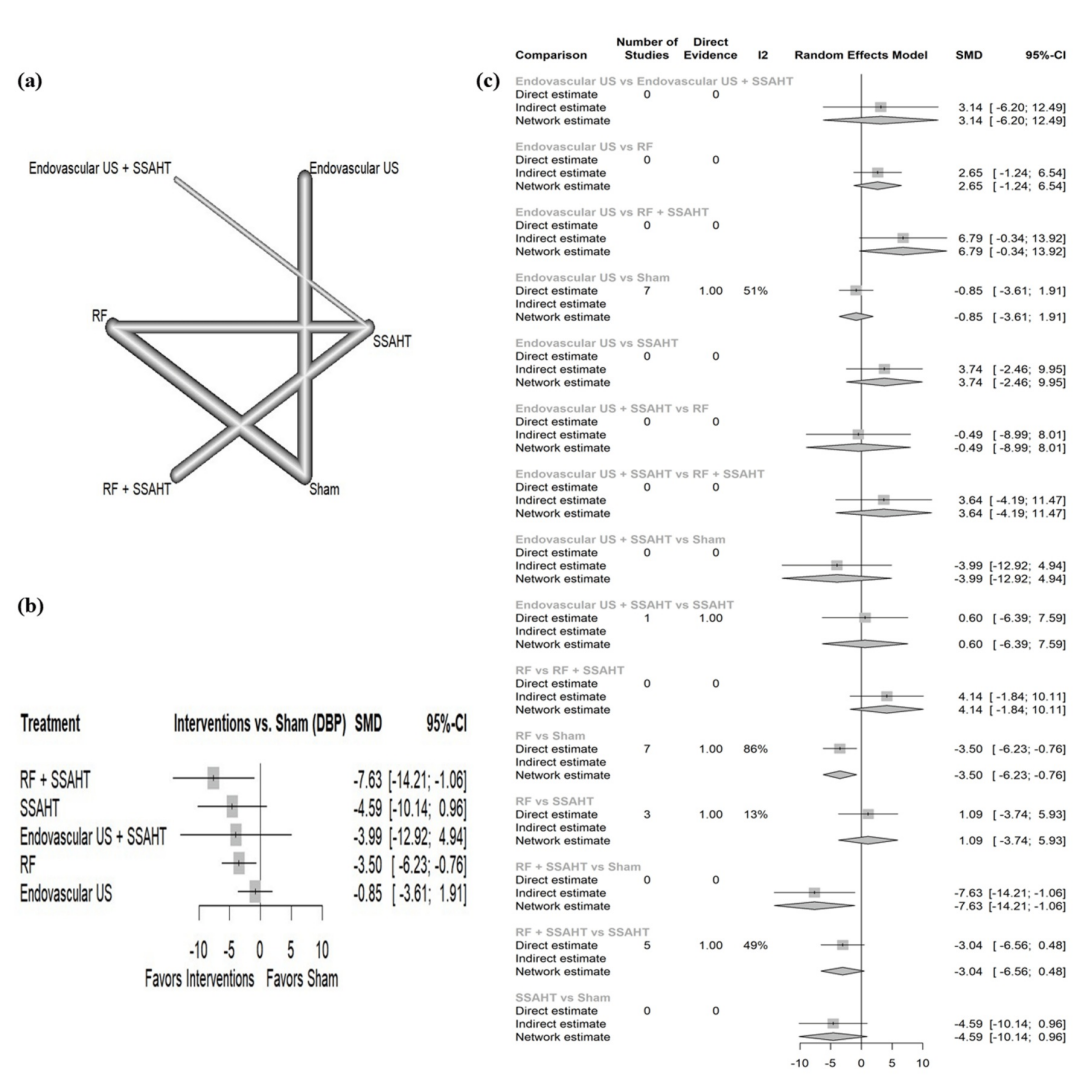
(a) Network geometry of DBP (nighttime). (b) Forest plot of change of DBP (nighttime) by different renal denervation interventions. (c) Direct versus indirect evidence of the change in DBP (nighttime) SSAHT: standardized stepped-care antihypertensive treatment; RF: radiofrequency; DBP: diastolic blood pressure; SMD: standardized mean difference

DBP nighttime: The network plot (Figure 5a) displays the direct comparisons between the different interventions included in the analysis. Figure 5b shows the results comparing various interventions against the sham procedure to affect DBP at nighttime. RF + SSAHT and RF alone have statistical significance with an SMD of -7.63 (95% CI: (-14.21; -1.06)) and SMD of -3.50 (95% CI: (-6.23; -0.76)), respectively. Figure 5c presents the percentage of direct and indirect evidence contributions for each comparison. Publication bias was assessed using a comparison-adjusted funnel plot (supplemental material 7); the funnel plot shows symmetry (p = 0.1907).

**Figure 6:**
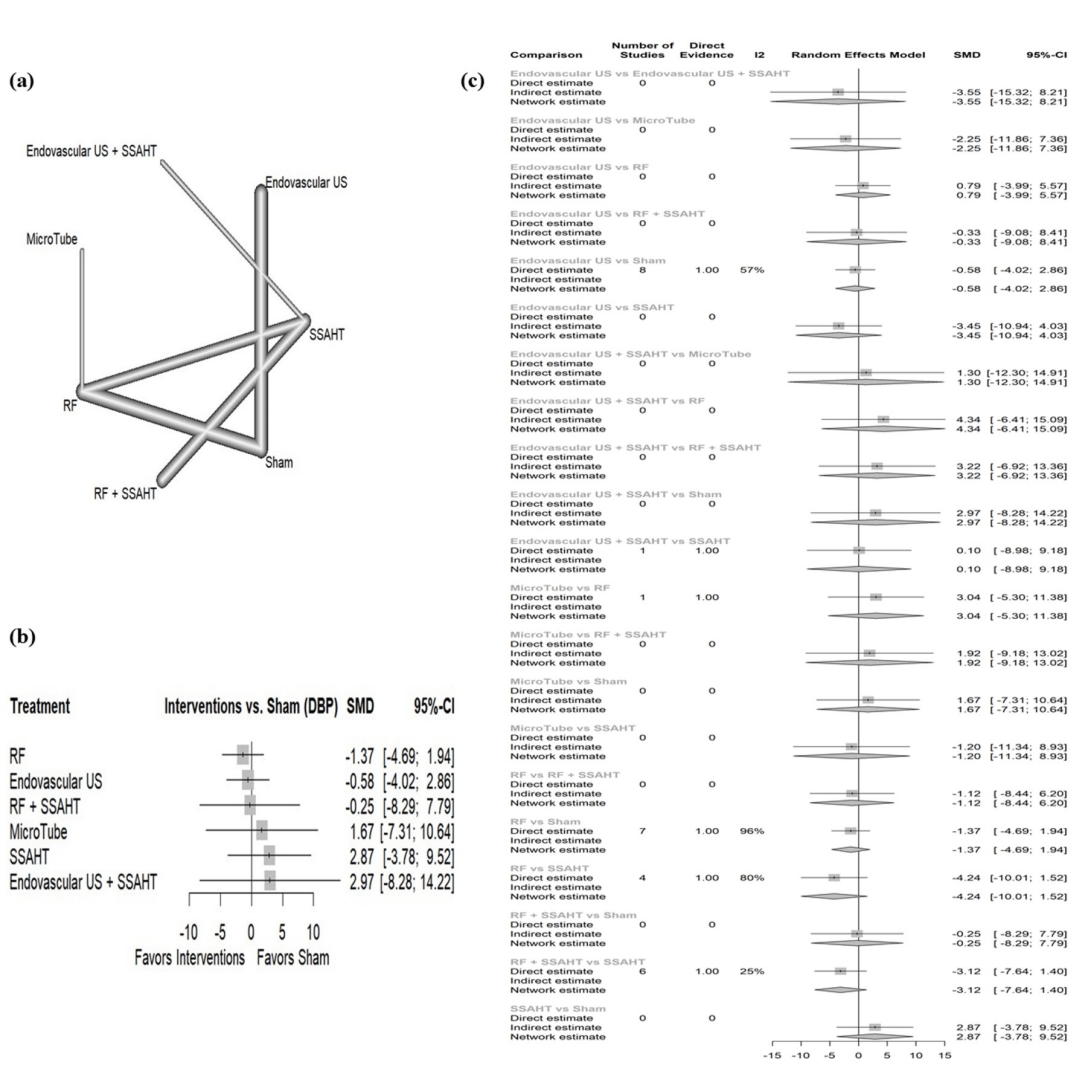
(a) Network geometry of DBP (office). (b) Forest plot of change of DBP (office) by different renal denervation interventions. (c) Direct versus indirect evidence of the change in DBP (office) SSAHT: standardized stepped-care antihypertensive treatment; RF: radiofrequency; DBP: diastolic blood pressure; SMD: standardized mean difference

DBP office: The network plot (Figure 6a) displays the direct comparisons between the different interventions included in the analysis. Figure 6b shows the results comparing various interventions against the sham procedure to affect DBP in the office. RF showed the largest reduction in DBP with a SMD of -1.37 (95% CI: (-4.69; -1.94)). All other interventions have no statistical significance. Figure 6c presents the percentage of direct and indirect evidence contributions for each comparison. Publication bias was assessed using a comparison-adjusted funnel plot (supplemental material 8); the funnel plot shows symmetry (p = 0.1907).

**Figure 7:**
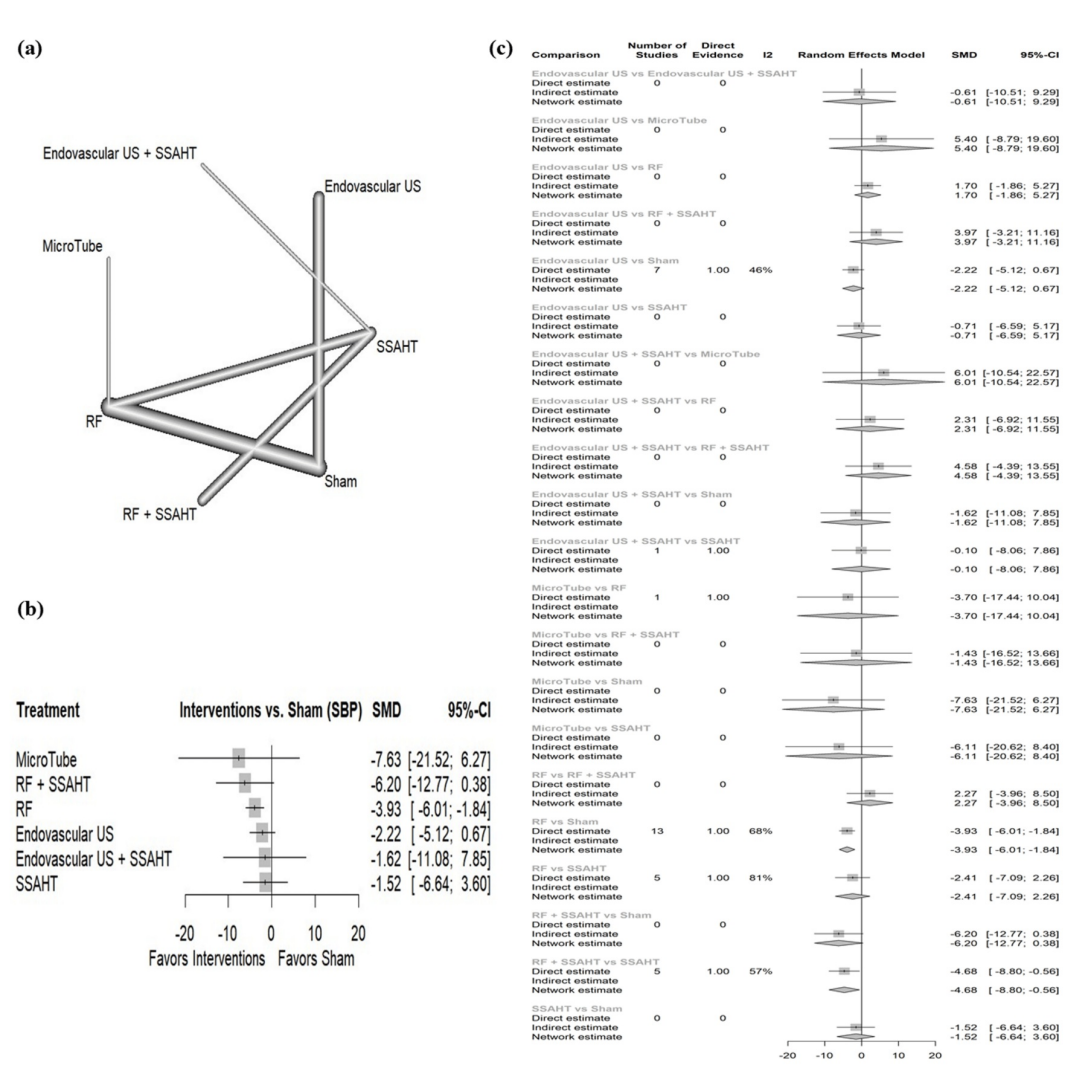
(a) Network geometry of SBP (24 hours). (b) Forest plot of change of SBP (24 hours) by different renal denervation interventions. (c) Direct versus indirect evidence of the change in SBP (24 hours) SSAHT: standardized stepped-care antihypertensive treatment; RF: radiofrequency; SBP: systolic blood pressure; SMD: standardized mean difference

SBP per 24 hours: The network plot (Figure 7a) displays the direct comparisons between the different interventions included in the analysis. Figure 7b shows the results comparing various interventions against the sham procedure to affect SBP per 24 hours. Microtube showed the largest reduction in SBP with an SMD of -7.63 (95% CI: (-21.52; 6.27)) with no statistical significance. The intervention RF alone demonstrated a statistically significant reduction in SBP (SMD: -3.93, 95% CI: (-6.01; -1.84)); other interventions show no statistical significance. Figure 7c presents the percentage of direct and indirect evidence contributions for each comparison. Publication bias was assessed using a comparison-adjusted funnel plot (supplemental material 9); the funnel plot shows symmetry (p = 0.5123).

**Figure 8:**
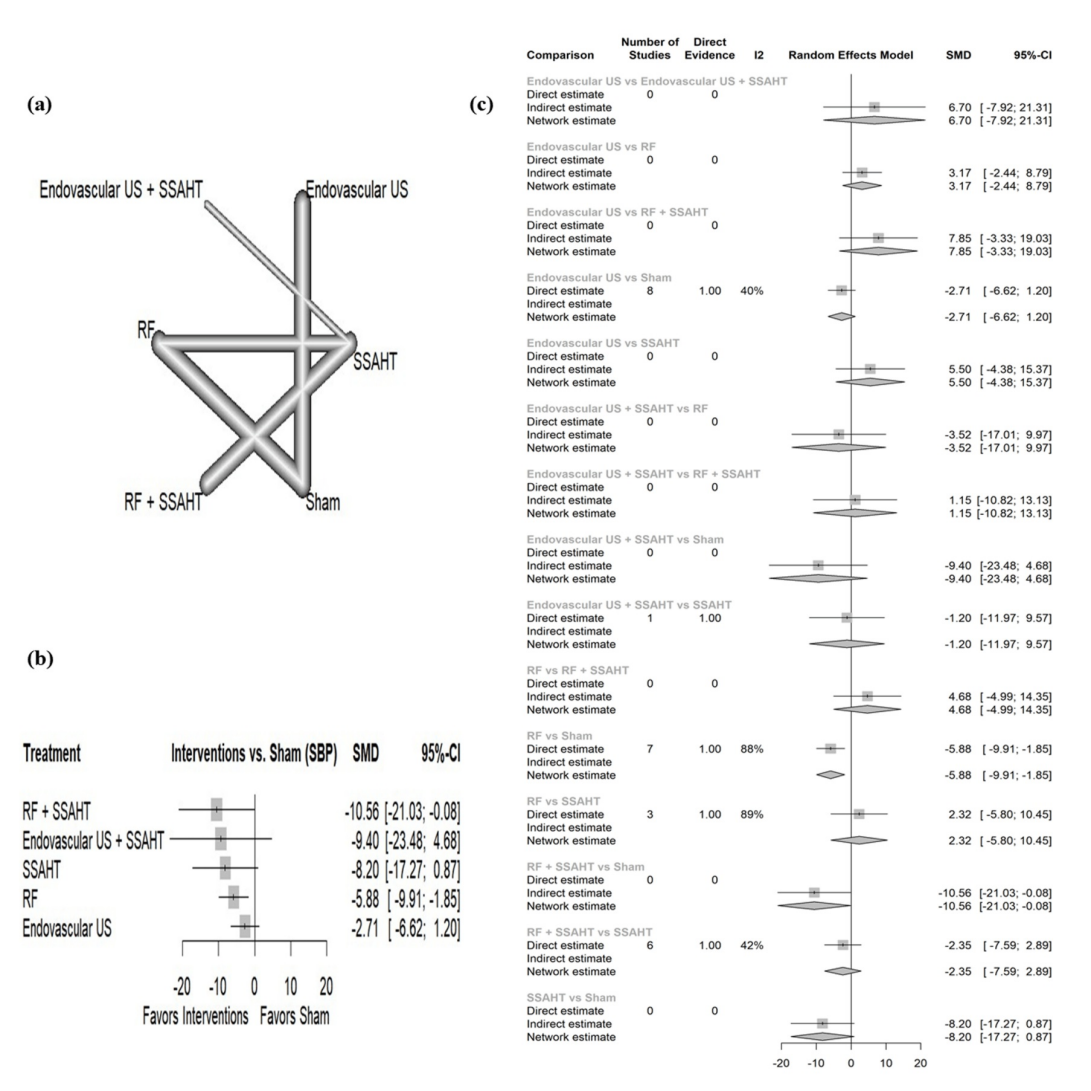
(a) Network geometry of SBP (daytime). (b) Forest plot of change of SBP (daytime) by different renal denervation interventions. (c) Direct versus indirect evidence of the change in SBP (daytime) SSAHT: standardized stepped-care antihypertensive treatment; RF: radiofrequency; SBP: systolic blood pressure; SMD: standardized mean difference

SBP daytime: The network plot (Figure 8a) displays the direct comparisons between the different interventions included in the analysis. Figure 8b shows the results comparing various interventions against the sham procedure to affect SBP during the daytime. RF + SSAHT and RF alone have statistical significance with an SMD of -10.56 (95% CI: (-21.03; -0.08)) and SMD of-5.88 (95% CI: (-9.91; -1.85)), respectively. Figure 8c presents the percentage of direct and indirect evidence contributions for each comparison. Publication bias was assessed using a comparison-adjusted funnel plot (supplemental material 10); the funnel plot shows symmetry (p = 0.1769).

**Figure 9:**
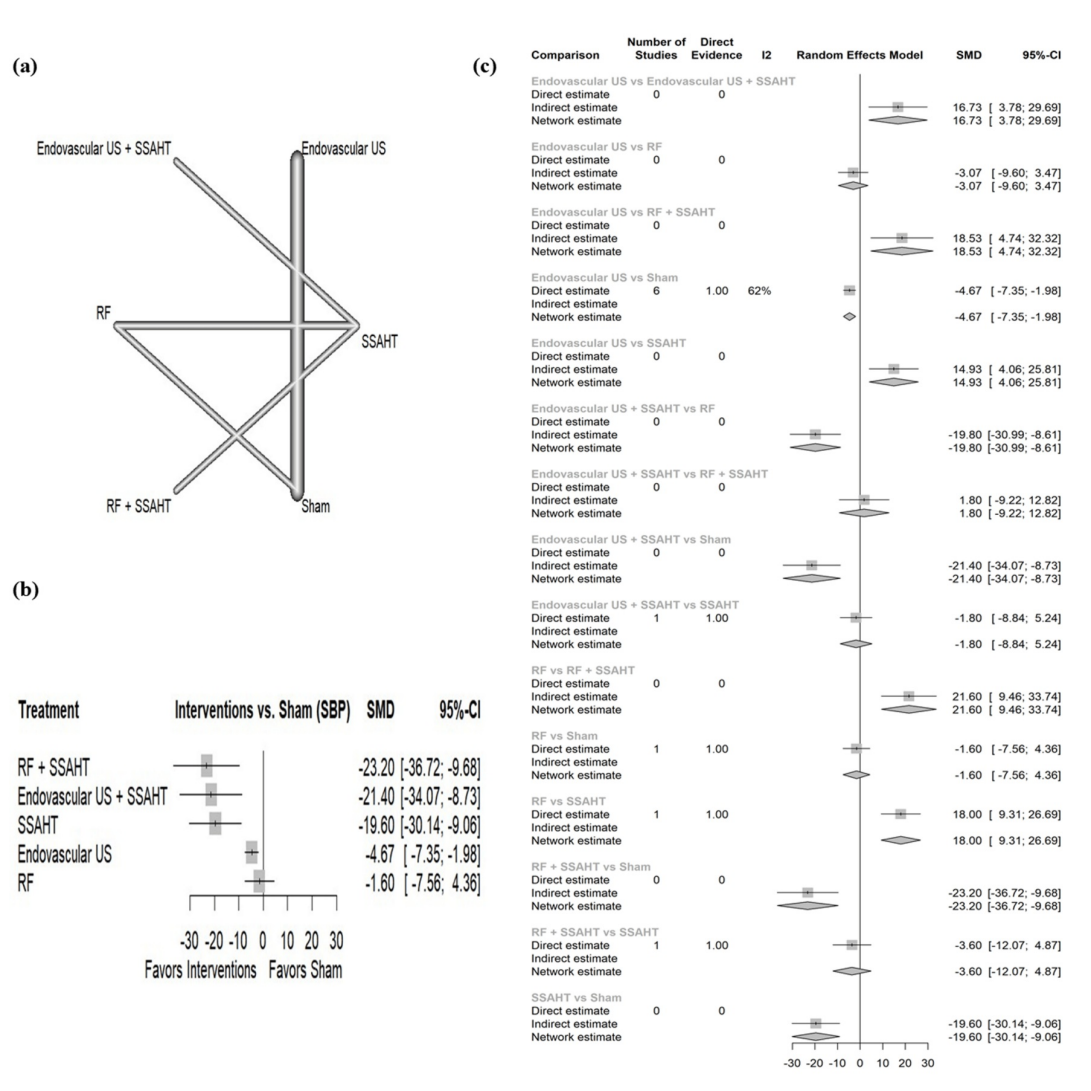
(a) Network geometry of SBP (home). (b) Forest plot of change of SBP (home) by different renal denervation interventions. (c) Direct versus indirect evidence of the change in SBP (home) SSAHT: standardized stepped-care antihypertensive treatment; RF: radiofrequency; SBP: systolic blood pressure; SMD: standardized mean difference

SBP home: The network plot (Figure 9a) displays the direct comparisons between the different interventions included in the analysis. Figure 9b shows the results comparing various interventions against the sham procedure to affect SBP at home. RF + SSAHT showed the largest reduction in SBP with an SMD of -23.20 (95% CI: (-36.72; -9.26)). SSAHT and endovascular US + SSAHT also show a statistically significant reduction in SBP with an SMD of -19.60 (95% CI: (-30.14; -9.06)) and SMD of -21.40 (95% CI: (-34.07; -8.73)), respectively. Figure 9c presents the percentage of direct and indirect evidence contributions for each comparison. Publication bias was assessed using a comparison-adjusted funnel plot (supplemental material 11); the funnel plot shows asymmetry (p = 0.0650).

**Figure 10:**
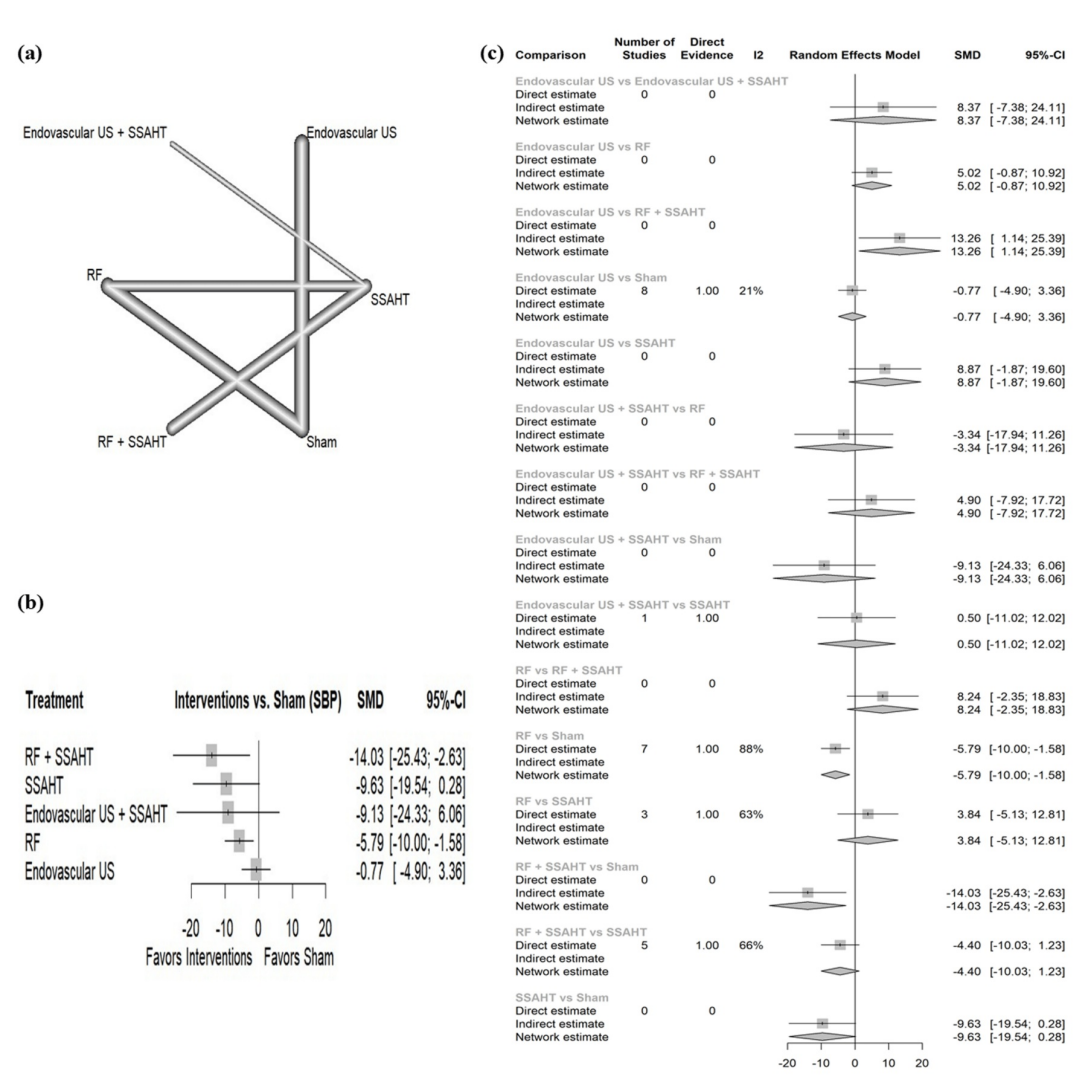
(a) Network geometry of SBP (nighttime). (b) Forest plot of change of SBP (nighttime) by different renal denervation interventions. (c) Direct versus indirect evidence of the change in SBP (nighttime) SSAHT: standardized stepped-care antihypertensive treatment; RF: radiofrequency; SBP: systolic blood pressure; SMD: standardized mean difference

SBP nighttime: The network plot (Figure 10a) displays the direct comparisons between the different interventions included in the analysis. Figure 10b shows the results comparing various interventions against the sham procedure to affect SBP at nighttime. RF + SSAHT showed the largest reduction in SBP with an SMD of -14.03 (95% CI: (-25.43; -2.63)). RF alone showed a statistically significant reduction in SBP with an SMD of -5.79 (95% CI: (-10.0; -1.58)). Figure 10c presents the percentage of direct and indirect evidence contributions for each comparison. Publication bias was assessed using a comparison-adjusted funnel plot (supplemental material 12); the funnel plot shows significant asymmetry (p = 0.0362).

**Figure 11:**
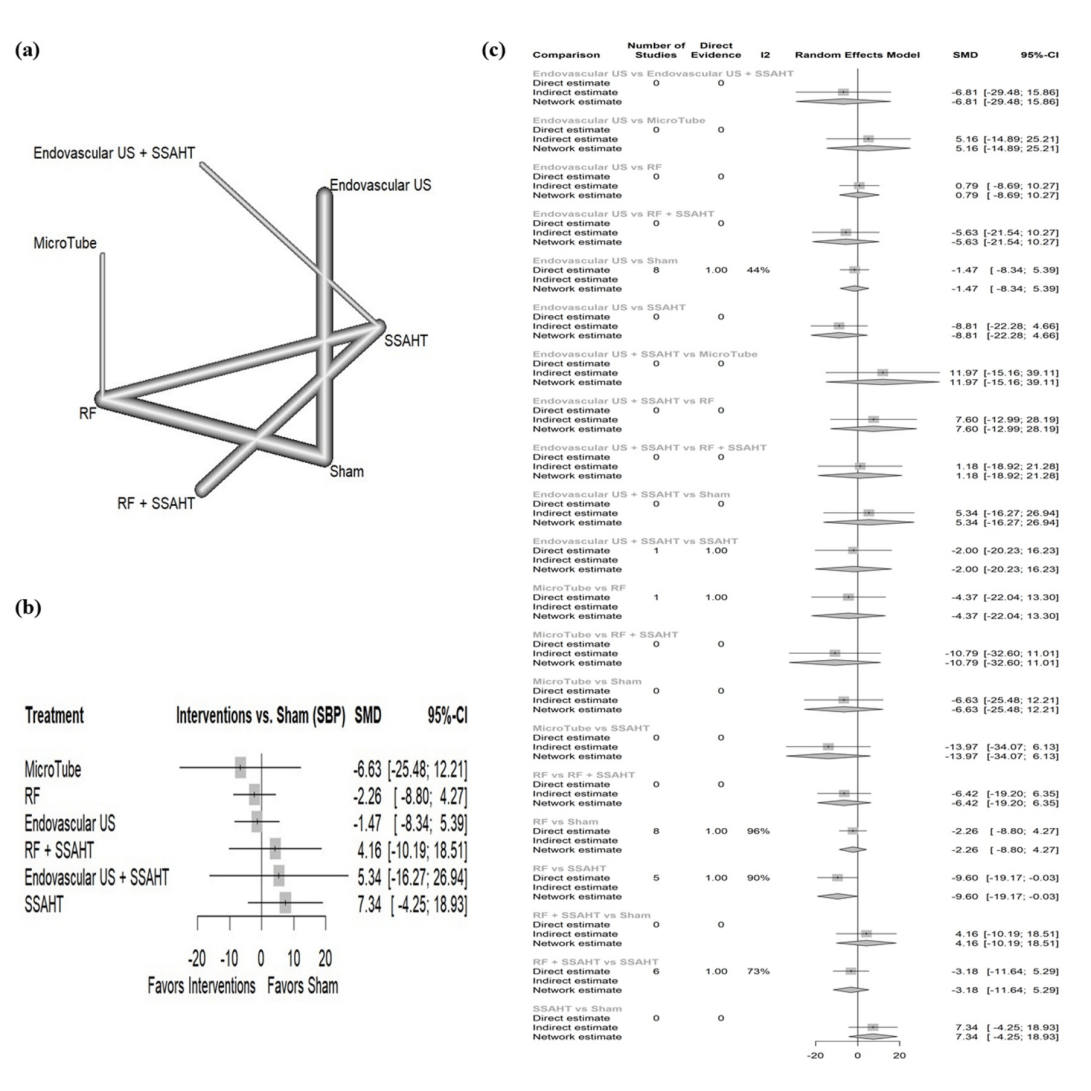
(a) Network geometry of SBP (office). (b) Forest plot of change of SBP (office) by different renal denervation interventions. (c) Direct versus indirect evidence of the change in SBP (office) SSAHT: standardized stepped-care antihypertensive treatment; RF: radiofrequency; SBP: systolic blood pressure; SMD: standardized mean difference

SBP office: The network plot (Figure 11a) displays the direct comparisons between the different interventions included in the analysis. Figure 11b shows the results comparing various interventions against the sham procedure to affect SBP in the office. Microtube showed the largest reduction with a SMD of -6.63 (95% CI: (-25.48; 12.21)). All interventions have no statistical significance. Figure 11c presents the percentage of direct and indirect evidence contributions for each comparison. Publication bias was assessed using a comparison-adjusted funnel plot (supplemental material 13); the funnel plot shows symmetry (p = 0.1127).

Assessment of Heterogeneity and Inconsistency

Our analysis, guided by the I^2 statistic, indicates there is significant heterogeneity across all network meta-analyses. The assessment for inconsistency through the node-splitting model indicated that there was no statistically significant difference between direct and indirect comparisons (p > 0.05).

Discussion

Summary of the Main Findings

In comparison to the other interventions, RF added to SSAHT was the best intervention to reduce DBP (24 hours), DBP (daytime), DBP (home), DBP (nighttime), SBP (daytime), SBP (home), and SBP (nighttime). On the other hand, the microtube was the best intervention to reduce the SBP (24 hours) and SBP (office) and RF alone was the best method to reduce DBP (office).

Significance of the Work

The finding of this study suggests that RDN may introduce a promising choice in the treatment of uncontrolled hypertension patients, especially in those who do not respond to medical therapy, although we still need more long-term trials that have larger sample sizes to evaluate the safety and efficacy of RDN catheters in a well-defined population to determine the preoperative predictors of the BP response to RDN.

Agreement and Disagreement With Previous Studies

In a previous systematic review, RDN was effective in reducing mean BP at six months in RH [5]. A previous network meta-analysis showed that RF has the best effect on reducing BP compared to other interventions [28].

Our meta-analysis shows significant benefits of RDN in reducing BP, but it differs from the previous studies in comparing different RDN modalities and their effect on BP. RF added to SSAHT was the most potent method compared to others to reduce BP outcomes in patients with uncontrolled RH.

Strength Points and Limitations

The studies included in our meta-analysis had several limitations. First, the follow-up periods were generally short. Second, there was no clear evidence to confirm whether complete RDN was achieved. Third, the sample sizes in the trials were relatively small. Last, most studies included the use of medical treatment in addition to the intervention.

Our network meta-analysis has the advantage of comparing multiple RDN procedures to identify the best intervention. One of our study limitations was the presence of significant heterogeneity across all network meta-analyses; because of the difference between trials in baseline participants` characteristics, population of studies, follow-up duration, and the use of anti-hypertensive medications different in type and number in most of the trials. Another limitation is that our study did not include an analysis of safety outcomes across RDN interventions; because the safety profile has variations according to the RDN catheter type used in the procedure.

Ongoing Studies and Implications for Clinical Practice

While early clinical trials showed mixed results [18,29], more recent trials have shown that with improved patient selection and procedural techniques, RDN can get significant reductions in blood pressure (BP) [23-25]. The ongoing studies are about to provide more information about the long-term efficacy and safety of RDN. Many studies are now trying to know more about patient selection, ideal procedural techniques, and long-term BP reduction. These studies will help to improve our knowledge of the RDN mechanisms and of the patient subgroup that will benefit most from intervention. Recent guidelines acknowledged the RDN as an adjunctive treatment for hypertension and recognized its efficacy in reducing BP over 24 hours.

## Conclusions

Our systematic review and network meta-analysis found that RF added to SSAHT was the most potent method compared to others to reduce BP outcomes in patients with uncontrolled or resistant hypertension. However, there are still a lot of questions about the long-term safety, effectiveness, and wider cardiovascular implications of this procedure. Further trials are required to establish the function of RDN in hypertension therapy and to identify the patient categories most likely to benefit from this intervention.

## Figures and Tables

**Figure 12:**
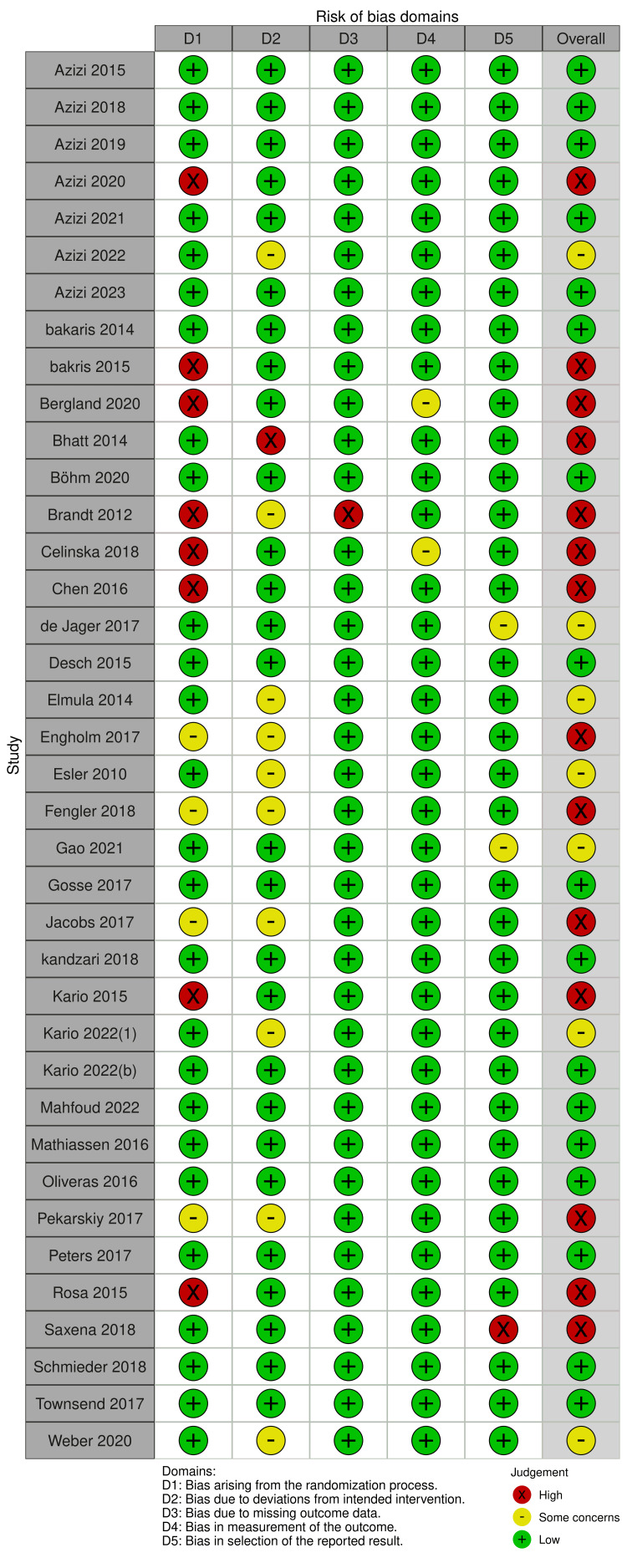
ROB graph Reference: [17,18,20-25,29-58] ROB: risk of bias

**Figure 13:**
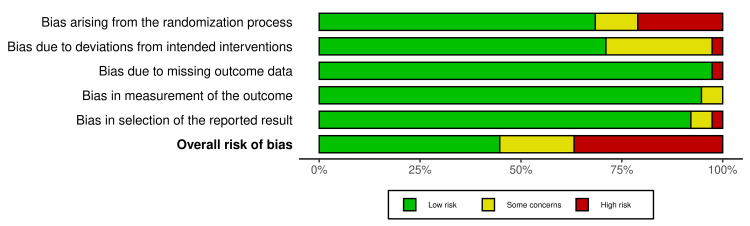
ROB summary ROB: risk of bias

**Figure 14:**
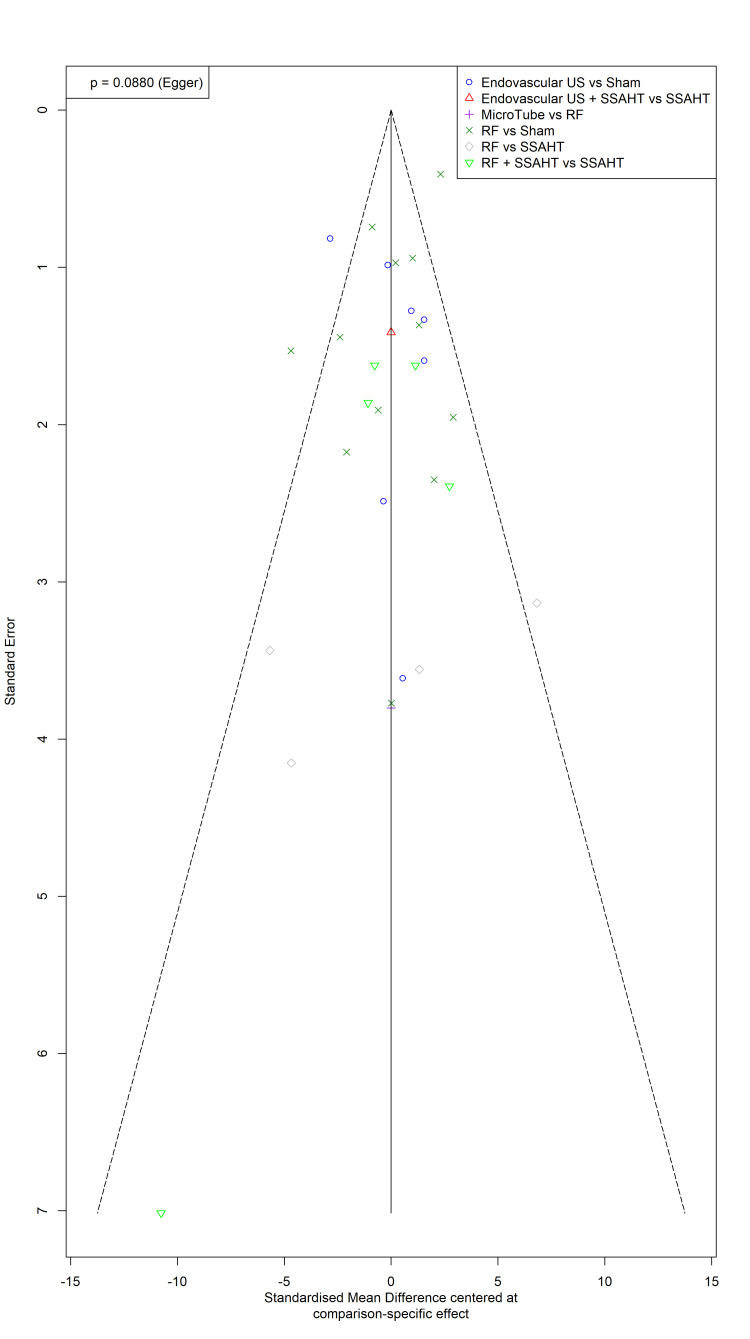
Funnel plot publication bias of DBP 24 hours DBP: diastolic blood pressure; SSAHT: standardized stepped-care antihypertensive treatment; RF: radiofrequency

**Figure 15:**
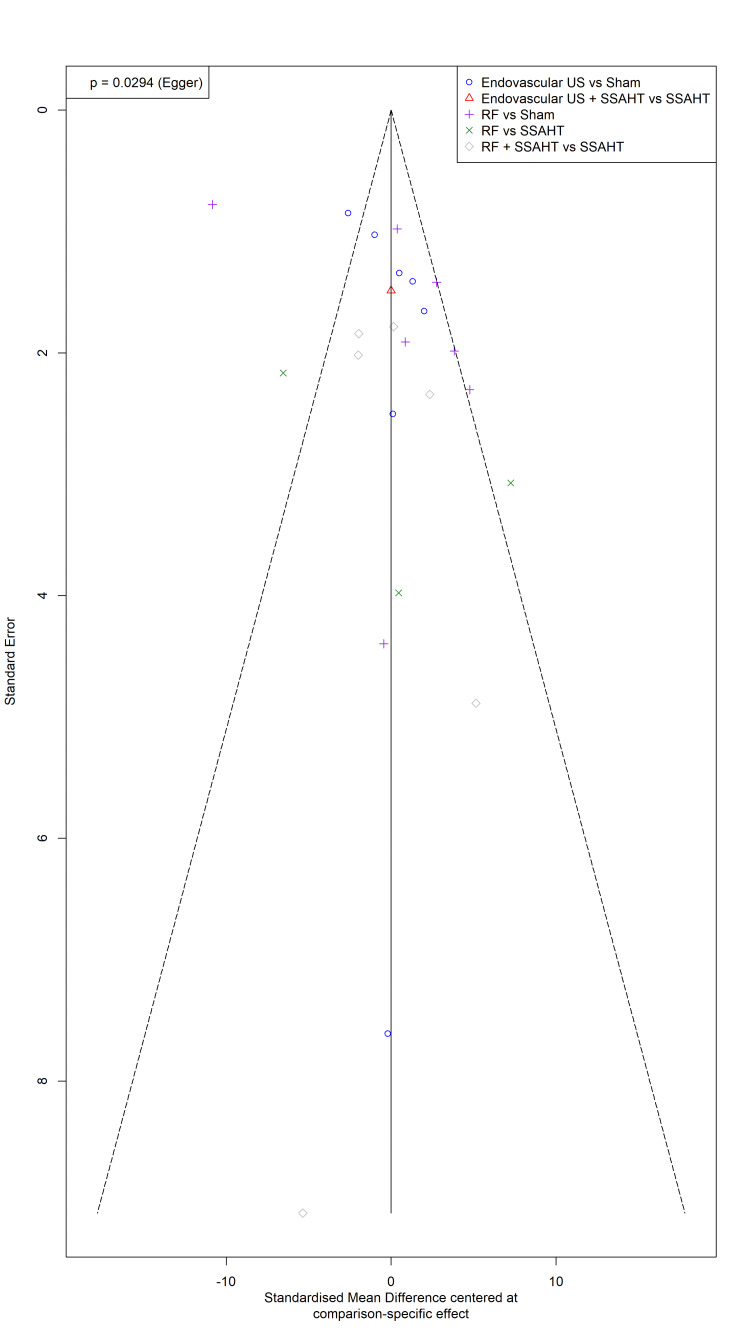
Funnel plot publication bias of DBP daytime DBP: diastolic blood pressure; SSAHT: standardized stepped-care antihypertensive treatment; RF: radiofrequency

**Figure 16:**
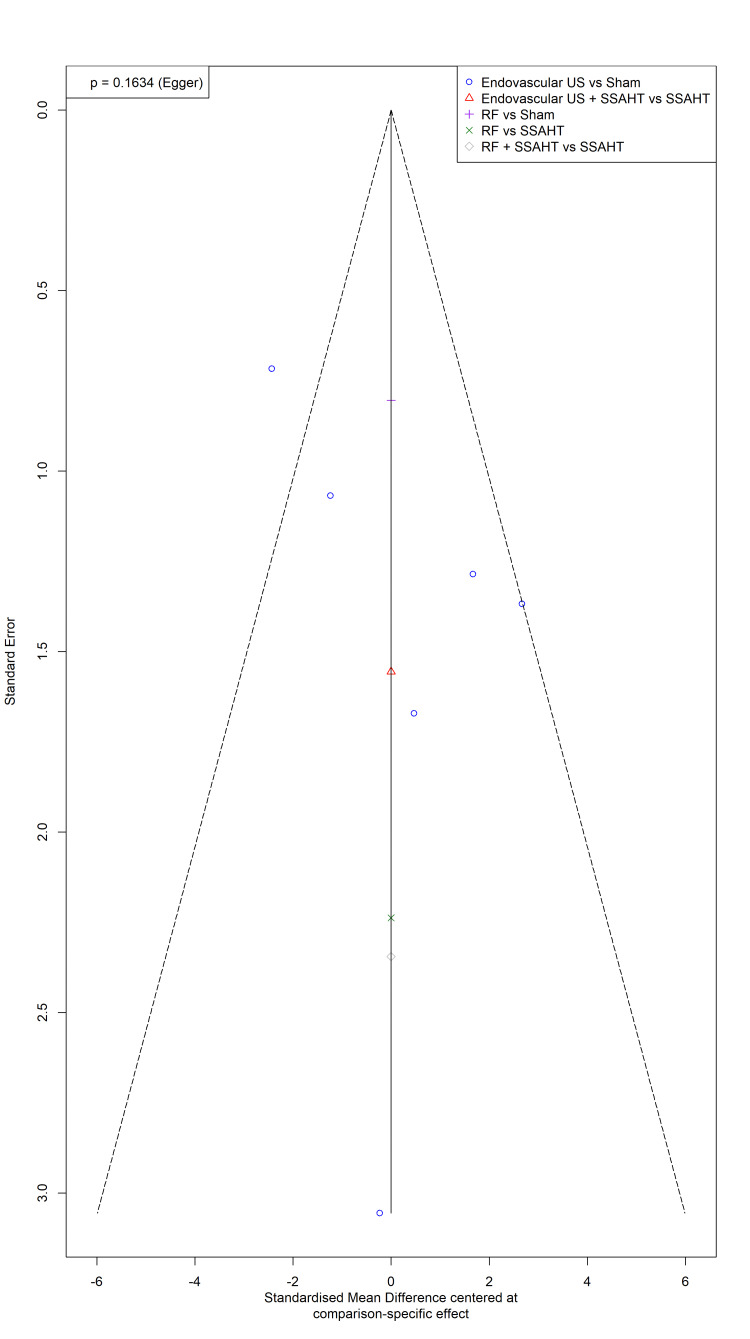
Funnel plot publication bias of DBP home DBP: diastolic blood pressure; SSAHT: standardized stepped-care antihypertensive treatment; RF: radiofrequency

**Figure 17:**
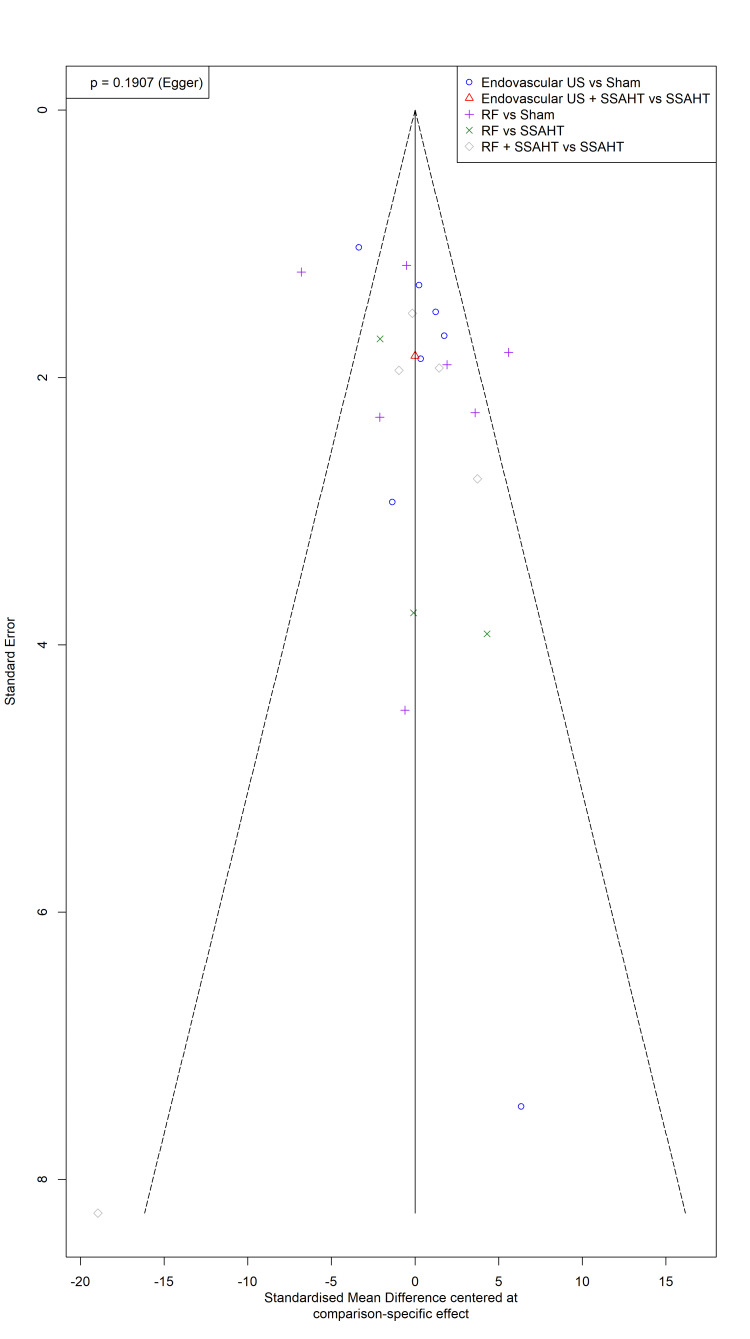
Funnel plot publication bias of DBP nighttime DBP: diastolic blood pressure; SSAHT: standardized stepped-care antihypertensive treatment; RF: radiofrequency

**Figure 18:**
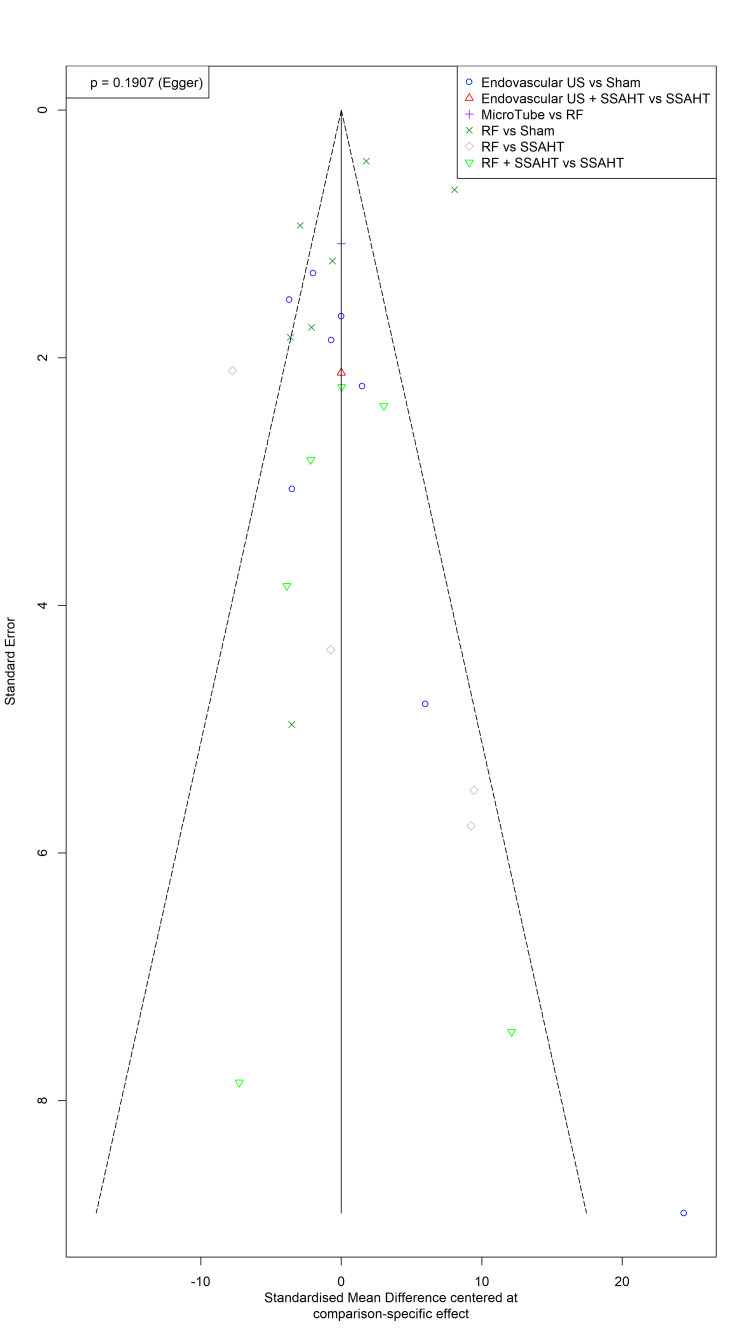
Funnel plot publication bias of DBP office DBP: diastolic blood pressure; SSAHT: standardized stepped-care antihypertensive treatment; RF: radiofrequency

**Figure 19:**
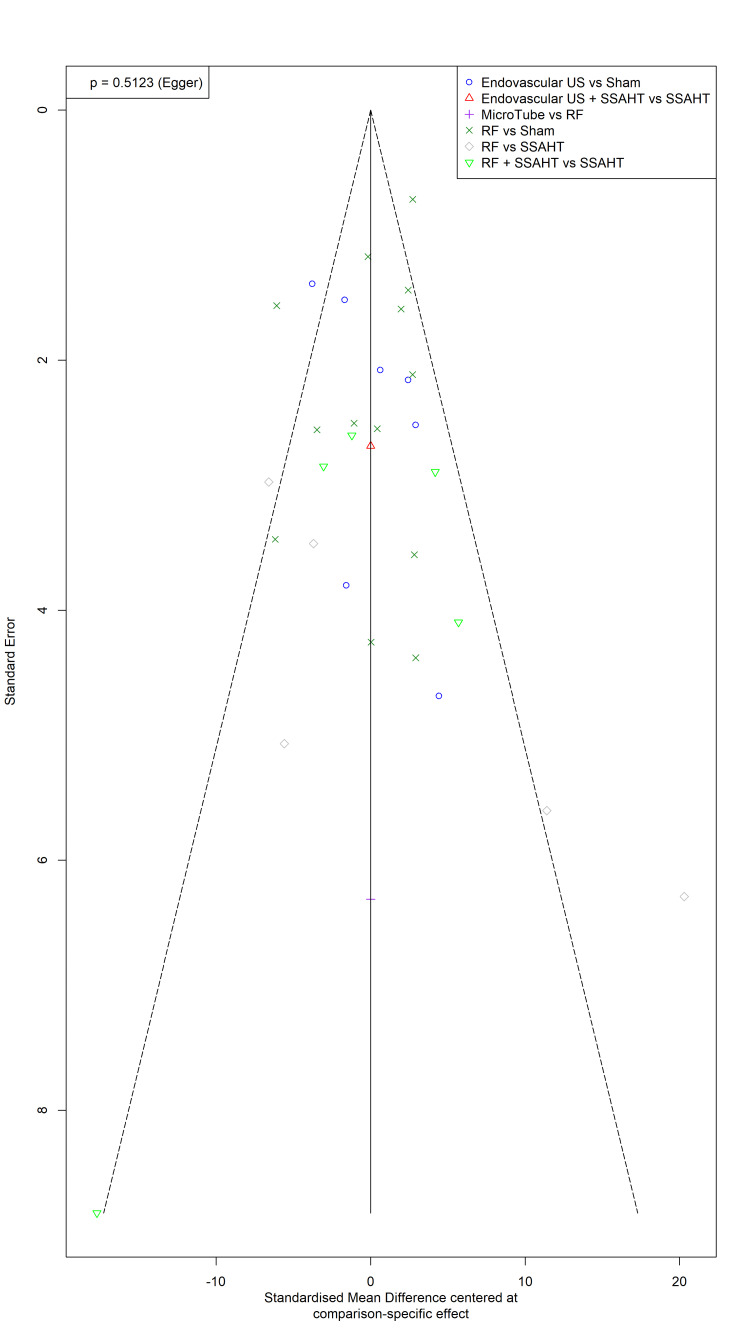
Funnel plot publication bias of SBP 24 hours SBP: systolic blood pressure; SSAHT: standardized stepped-care antihypertensive treatment; RF: radiofrequency

**Figure 20:**
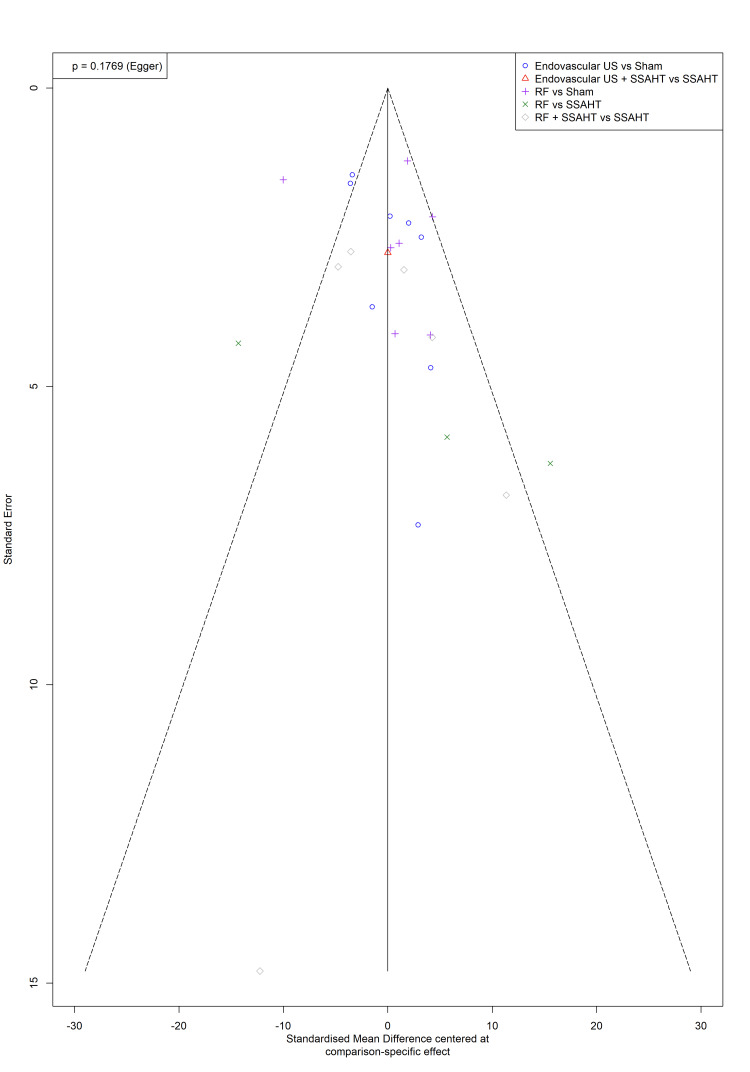
Funnel plot publication bias of SBP daytime SBP: systolic blood pressure; SSAHT: standardized stepped-care antihypertensive treatment; RF: radiofrequency

**Figure 21:**
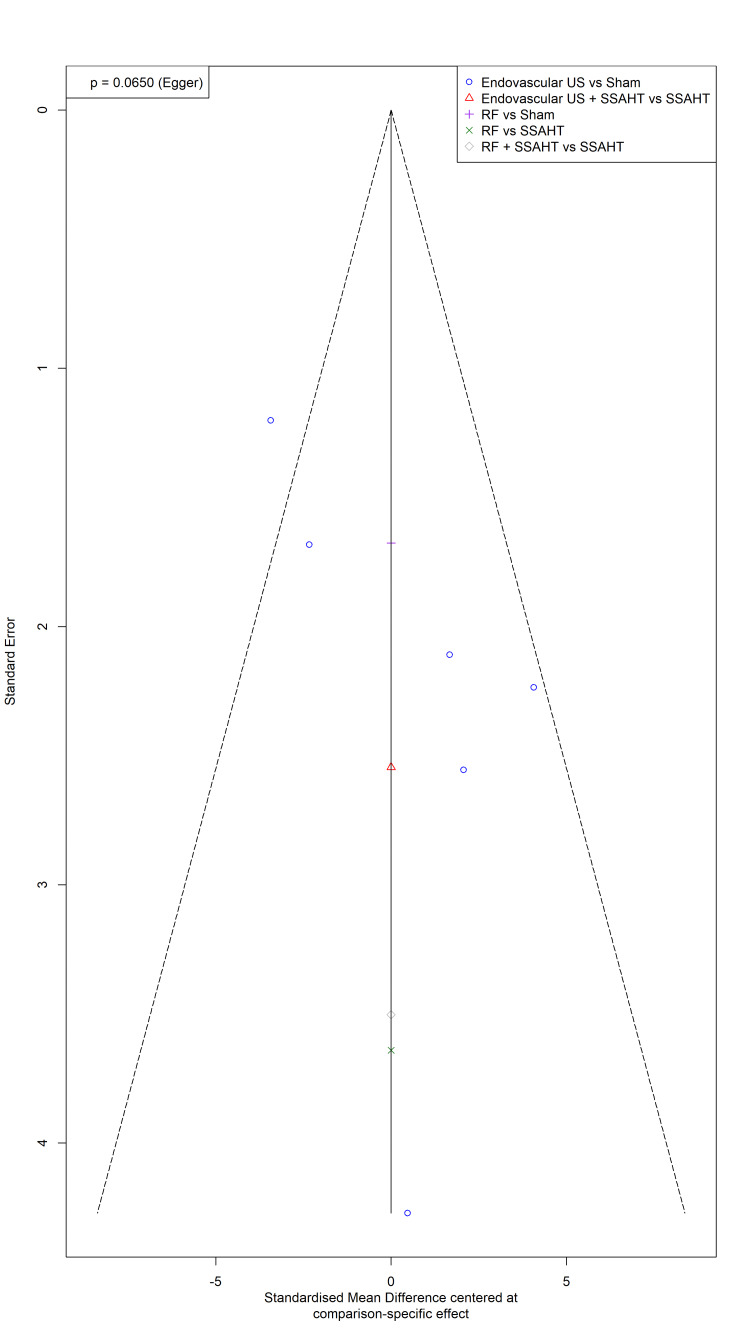
Funnel plot publication bias of SBP home SBP: systolic blood pressure; SSAHT: standardized stepped-care antihypertensive treatment; RF: radiofrequency

**Figure 22:**
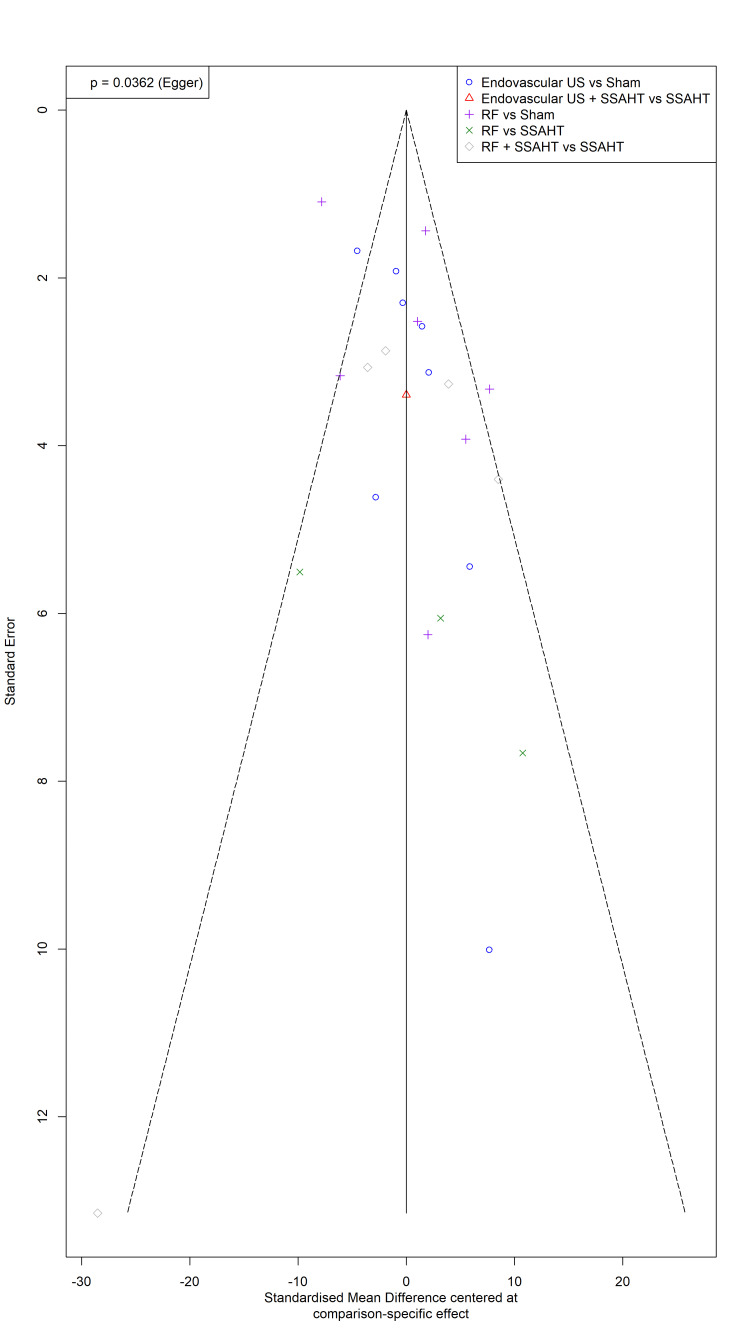
Funnel plot publication bias of SBP nighttime SBP: systolic blood pressure; SSAHT: standardized stepped-care antihypertensive treatment; RF: radiofrequency

**Figure 23:**
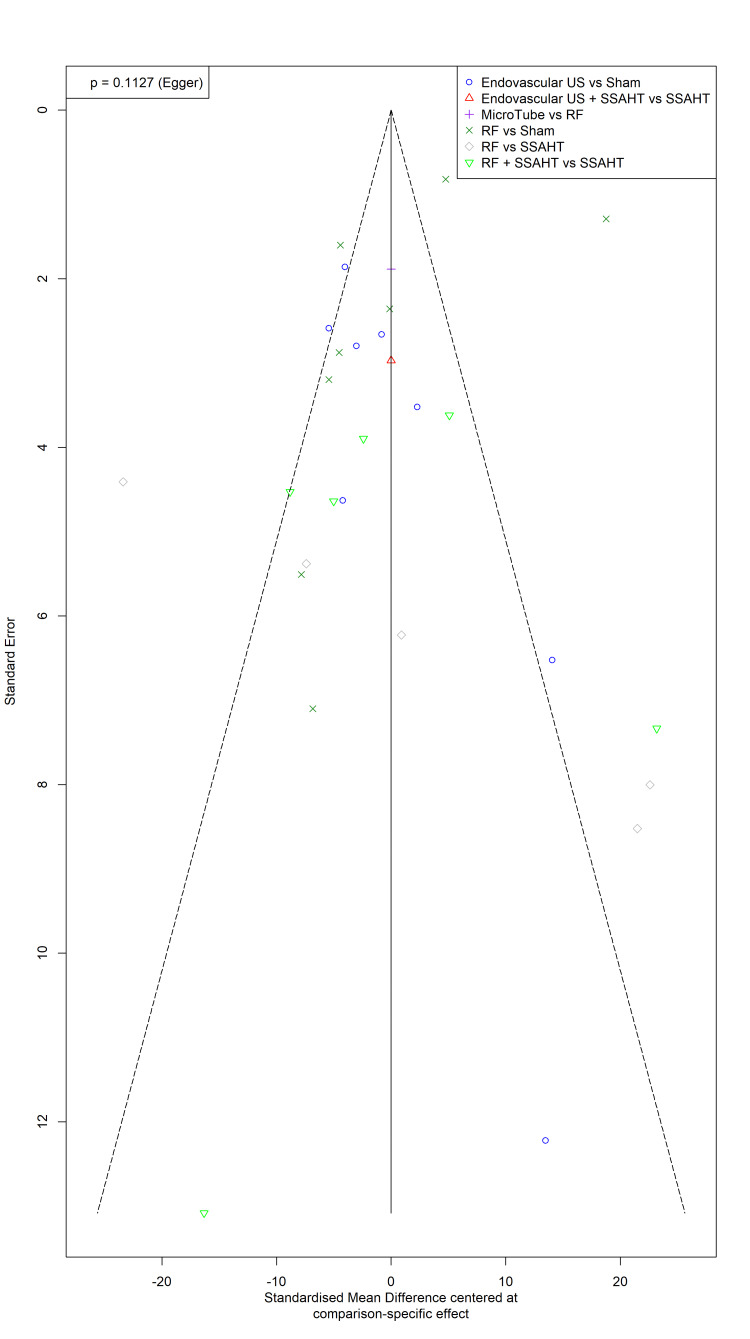
Funnel plot publication bias of SBP office SBP: systolic blood pressure; SSAHT: standardized stepped-care antihypertensive treatment; RF: radiofrequency

## Appendices

Supplemental material 1

**Table 1:** Summary of included studies and baseline participants characteristics AHT: antihypertensive treatment; SBP: systolic blood pressure; DBP: diastolic blood pressure; GFR: glomerular filtration rate; SSAHT: standardized stepped-care antihypertensive treatment; RDN: renal denervation

Study ID	DOI	Location	Study design	Population	Inclusion criteria	Intervention	Comparator	Outcome	Follow-up duration	results	sample size	Group	Sample size	Age mean (SD)	Sex (%)	BMI (kg/m^2^)	No. of AHT at baseline	No. of AHT in follow-up	Type of AHT (ACE I, CCB, Diuretics, BB, Spironolactone)	Race	HR mean (SD)	DBP 24 hours mean (SD)	SBP 24 hours mean (SD)	Type of renal disease	Type of Sham	GFR mean (SD)	creatinine level mean (SD)
Azizi 2015 [30]	http://dx.doi.org/10.1016/S0140-6736(14)61942-5	France	Multi-center, open label, randomized controlled trial with blinded endpoint	Eligible patients were men or women aged 18–75 years with resistant hypertension	Eligible patients were men or women aged 18–75 years referred for resistant hypertension	Radiofrequency-based renal denervation added to a standardized stepped-care antihypertensive treatment (SSAHT) (renal denervation group)	Standardized stepped-care antihypertensive treatment only (SSAHT) alone	The mean change in daytime systolic blood pressure; Secondary endpoints included mean changes in all other blood pressure variables from baseline to 6 months assessed by ambulatory, home, and office blood pressure	6 months	The mean change in daytime ambulatory systolic blood pressure at 6 months was −15·8 mm Hg (95% CI −19·7 to −11·9) in the renal denervation group and −9·9 mm Hg (−13·6 to −6·2) in the group receiving SSAHT alone, a baseline-adjusted difference of −5·9 mm Hg (−11·3 to −0·5; p=0·0329).	106	RDN+SSAHT	53	55·2 (10·8)	34 (64·2%)	30·7(4·8)	NR		Amlodipine 5 mg and 10 mg, Irbesartan 300 mg, Indapamide 1.5mg, Ramipril 10mg	42 (79.2%) Caucasian	72.6 (10.7)	90.2 (15.3)	151.6 (16.3)		SSAHT	88 (24)	85 (23)
												SSAHT only	53	55·2 (10·1)	32 (60·4%)	29·7(4·5)				41 (77.4%) Caucasian	74.1 (11.6)	88.8 (10.6)	146.8 (15.2)			90 (24)	83 (25)
Azizi 2018 [22]	https://doi.org/10.1016/S0140-6736(18)31082-1	USA & Europe	Multicenter, international, single-blind, randomized, sham-controlled trial	Patients with combined systolic–diastolic hypertension aged 18–75 years were eligible if they had ambulatory blood pressure greater than or equal to 135/85 mm Hg and less than 170/105 mm Hg after a 4-week discontinuation of up to two antihypertensive medications and had suitable renal artery anatomy. Also, eGFR of greater than or equal 40 ml	Eligible patients were men or women aged 18–75 years with combined systolic–diastolic hypertension that was either uncontrolled on zero to two antihypertensive medications (average seated office systolic and diastolic blood pressure of ≥140/90 mm Hg, but <180/110 mm Hg) or controlled on one to two antihypertensive medications (average seated office blood pressure <140/90 mm Hg). Patients also needed to have an estimated glomerular filtration rate (eGFR) of greater than or equal to 40 mL/ min per 1·73 m² (based on the Modification of Diet in Renal Disease formula) and no history of cardiovascular or cerebrovascular events.	Endovascular ultrasound renal denervation	sham	The change in daytime ambulatory systolic blood pressure at 2 months; Secondary efficacy endpoints specified for hierarchical testing were change in average 24-h ambulatory systolic blood pressure, average 24-h ambulatory diastolic blood pressure, average night-time ambulatory systolic blood pressure, and average night-time ambulatory diastolic blood pressure at 2 months, in this order. Additional specified secondary efficacy endpoints included change in daytime ambulatory diastolic blood pressure, office and home systolic and diastolic blood pressures, ambulatory and office heart rates,	2 months	The reduction in daytime ambulatory systolic blood pressure was greater with renal denervation (–8·5 mm Hg, SD 9·3) than with the sham procedure (–2·2 mm Hg, SD 10·0; baseline-adjusted difference between groups: –6·3 mm Hg, 95% CI –9·4 to –3·1, p=0·0001). No major adverse events were reported in either group.	146	URDN	74	54·4 (10·2)	46 (62%)	29·9 (5·9)	0: 12 (16%) 1: 33 (45%) 2: 28 (38%) 3: 1 (1%)	NR	Renin angiotensin system blockers (Angiotensin converting enzyme inhibitor, Angiotensin receptor blocker, Direct renin inhibitor), Calcium channel blocker, Diuretic, Beta-blocker, Alpha-1 receptor blocker, spironolactone)	White: 60 (81%) Black 12 (16%) other 2 (3%)	72 (12.1)	87.3 (5)	142.6 (8.1)		Standard AHT medications	84.7 (16.2)	NR
												sham	72	53·8 (10·0)	39 (54%)	29·0 (5·0)	0: 16 (22%) 1: 28 (39%) 2: 27 (38%) 3: 1 (1%)			"White: 52 (72%) Black 13 (18%) other 7 (10%)"	72.6 (12.3)	88.6 (5.7)	143.8 (10.4)			83.2 (16.1)	
Azizi 2019 [23]	10.1161/CIRCULATIONAHA.119.040451	USA & Europe	Multicenter, international, randomized, blinded, sham-controlled trial.	Patients with combined systolic–diastolic hypertension aged 18–75 years were eligible if they had ambulatory blood pressure greater than or equal to 135/85 mm Hg and less than 170/105 mm Hg after a 4-week discontinuation of up to two antihypertensive medications and had suitable renal artery anatomy.	Eligible patients were aged 18 to 75 years with office systolic and diastolic BP (DBP) ≥140/90 mmHg but <180/110 mmHg on 0 to 2 hypertensive medications (uncontrolled) or with office BP <140/90 mmHg (controlled) on 1 to 2 antihypertensive medications, had no history of cardiovascular or cerebrovascular events, and had an estimated glomerular filtration rate (eGFR) ≥40 mL/min/1.73 m^2^ (Modification of Diet in Renal Disease formula).	Endovascular ultrasound renal denervation	sham	The baseline and covariate-adjusted change in daytime ambulatory SBP at 6 months. Other efficacy end points included baseline and covariate-adjusted change at 6 months in all other ambulatory, home, and office BP measurements and in ambulatory heart rate.	6 months	Despite less intensive standardized stepped-care antihypertensive treatment, RDN reduced daytime ambulatory systolic BP to a greater extent than sham -18.1±12.2 versus -15.6±13.2 mmHg, respectively; difference adjusted for baseline BP and number of medications: −4.3 mmHg, 95% confidence interval, -7.9 to -0.6, P=0.024).	140	URDN	69	54·1(10.2)	43(62.3%)	29.9 (5.9)	0: 12 (17.4%) 1: 30 (43.5%) 2: 26 (37.7%) 3: 1 (1.4%)	0: 24 (34.8%) 1: 29 (42%) 2: 12 (17.4%) 3: 4 (5.8%)	Renin angiotensin system blockers (Angiotensin converting enzyme inhibitor, Angiotensin receptor blocker, Direct renin inhibitor), Calcium channel blocker, Diuretic, Beta-blocker, Alpha-1 receptor blocker, spironolactone)	White: 56 (81.2%) Black 11 (15.9%) other 2 (2.9%)	72.6 (11.8)	87.3 (4.9)	142.4 (8.2)		Standard AHT medications	84.2 (15.7)	NR
												Sham	71	53.8 (10.1)	39 (54.9%)	29.0 (5.0)	0: 16 (22.5%) 1: 28 (39.4%) 2: 26 (36.6%) 3: 1 (1.4%)	0: 11 (15.5%) 1: 34 (47.9%) 2: 19 (26.8%) 3: 7 (9.9%)		"White: 51 (71.8%) Black 13 (18.3%) other 7 (9.9%)"	73.1 (12.4)	88.5 (5.7)	143.7 (10.4)			82.9 (16.1)	
Azizi 2020 [31]	https://doi.org/10.1016/j.jcin.2020.09.054	USA & Europe	Multicenter, international, randomized, unblinded phase, sham-controlled trial.	Patients with combined systolic–diastolic hypertension aged 18–75 years were eligible if they had ambulatory blood pressure greater than or equal to 135/85 mm Hg and less than 170/105 mm Hg after a 4-week discontinuation of up to two antihypertensive medications and had suitable renal artery anatomy.	Subjects who meet any one of the following criteria should be given consideration for inclusion in the Solo Cohort: Appropriately signed and dated informed consent Age ≥18 and ≤75 years at time of consent; Documented history of essential hypertension; Either, average seated office BP <180/110 mmHg at screening visit (V0) while on a stable regimen of 1 or 2 antihypertensive medications for at least 4 weeks prior to consent or, average seated office BP ≥ 140/90 mmHg <180/110 mmHg despite lifestyle measures on no antihypertensive medications; Documented daytime ABP ≥135/85 mmHg and <170/105 mmHg after 4-week washout/run-in period Suitable renal anatomy compatible with the renal denervation procedure and documented by renal CTA or MRA of good quality performed within one year prior to consent (a CTA or MRA will be obtained in patients without a recent (≤1 year) renal imaging; Able and willing to comply with all study procedures	Ultrasound renal denervation in presence of antihypertensive medications	Sham	Outcomes at 12 months included medication burden, change in daytime ambulatory systolic BP (dASBP) and office or home systolic BP (SBP), visit-to-visit variability in SBP, and safety.	12 months	Despite unblinding, the BP-lowering effect of RDN was maintained at 12 months with fewer prescribed medications compared with sham.	132	URDN	65	54.3 (10.5)	43 (66.1%)	29.6 (5.7)	NA	NA	NA	NA	NA	NA	NA	NA	NA	NA	NA
												sham	67	54.1 (10.3)	35 (52.2%)	28.7 (4.9)	NA	NA	NA	NA	NA	NA	NA	NA	NA	NA	NA
Azizi 2021 [25]	10.1016/S0140-6736(21)00788-1	USA & Europe	Randomized, multicentre, single-blind, sham-controlled trial	Patients aged 18-75 years with office blood pressure of at least 140/90 mm Hg despite three or more antihypertensive medications including a diuretic	Men or women aged 18–75 years with resistant hypertension defined as seated office blood pressure of at least 140 mm Hg systolic and 90 mm Hg diastolic despite a stable regimen of three or more antihypertensive medications including a diuretic, and an estimated glomerular filtration rate of at least 40 mL/min per 1·73 m²	Endovascular ultrasound renal denervation	Sham	The change in daytime ambulatory systolic blood pressure at 2 months in the intention-to-treat population. Secondary efficacy endpoints specified for hierarchical testing at 2 months were change in 24-h ambulatory systolic and diastolic blood pressures, night- time ambulatory systolic and diastolic blood pressures, and daytime ambulatory diastolic blood pressure.	2 months	Compared with a sham procedure, ultrasound renal denervation reduced blood pressure at 2 months in patients with hypertension resistant to a standardized triple combination pill. If the blood pressure lowering effect and safety of renal denervation are maintained in the long term, renal denervation might be an alternative to the addition of further antihypertensive medications in patients with resistant hypertension.	136	URDN	69	52·3 (7·5)	56 (81%)	32·8 (5·7)	NA	NA	NA	NA	NA	NA	NA	NA	NA	NA	NA
												Sham	67	52·8 (9·1)	53 (79%)	32·6 (5·4)	NA	NA	NA	NA	NA	NA	NA	NA	NA	NA	NA
Azizi 2022 [32]	https://doi.org/10.1001/jamacardio.2022.3904	USA & Europe	This randomized, sham-controlled, clinical trial with outcome assessors and patients blinded to treatment assignment	Patients aged 18–75 years with office blood pressure of at least 140/90 mm Hg despite three or more antihypertensive medications including a diuretic	Men or women aged 18–75 years with resistant hypertension defined as seated office blood pressure of at least 140 mm Hg systolic and 90 mm Hg diastolic despite a stable regimen of three or more antihypertensive medications including a diuretic, and an estimated glomerular filtration rate of at least 40 mL/min per 1·73 m²	Endovascular ultrasound renal denervation	Sham	Six-month change in medications, change in daytime ambulatory systolic BP, change in home systolic BP adjusted for baseline BP and medications, and safety.	6 months	In this study, in patients with RHTN initially randomly assigned to uRDN or a sham procedure and who had persistent elevation of BP at 2 months after the procedure, standardized stepped-care antihypertensive treatment escalation resulted in similar BP reduction in both groups at 6 months, with fewer additional medications required in the uRDN group.	129	URDN	65	51.9±7.5	53 (81.5%)	32.8±5.7	NA	NA	NA	NA	NA	NA	NA	NA	NA	NA	NA
												Sham	64	53.0±9.1	51 (79.7%)	32.7±5.5	NA	NA	NA	NA	NA	NA	NA	NA	NA	NA	NA
Azizi 2023 [33]	10.1001/jama.2023.0713	USA & Europe	Sham-controlled, randomized clinical trial with patients and outcome assessors blinded to treatment	Patients aged 18 years to 75 years with hypertension	Patients aged 18 years to 75 years with hypertension (seated office systolic BP [SBP] ≥140 mm Hg and diastolic BP [DBP] ≥90 mm Hg despite taking up to 2 antihypertensive medications) were eligible if they had an ambulatory SBP/DBP of 135/85 mm Hg or greater and an SBP/DBP less than 170/105 mm Hg after a 4-week washout of their medications. Patients with an estimated glomerular filtration rate of 40 mL/min/1.73 m^2 ^or greater and with suitable renal artery anatomy	Ultrasound renal denervation	Sham	The primary efficacy outcome was the mean change in daytime ambulatory SBP at 2 months. The primary safety composite outcome of major adverse events included death, kidney failure, and major embolic, vascular, cardiovascular, cerebrovascular, and hypertensive events at 30 days and renal artery stenosis greater than 70% detected at 6 months. The secondary outcomes included mean change in 24-hour ambulatory SBP, home SBP, office SBP, and all DBP parameters at 2 months. Tertiary outcomes included change at 2 months in night- time ambulatory measurements and ambulatory, home, and office heart rate measurements.	2 months	In patients with hypertension, ultrasound renal denervation reduced daytime ambulatory SBP at 2 months in the absence of antihypertensive medications vs a sham procedure without postprocedural major adverse events.	224	URDN	150	55.1 (9.9)	103 (68.7)	30.1 (5.2)	NA	NA	NA	NA	NA	NA	NA	NA	NA	NA	NA
												Sham	74	54.9 (7.9)	57 (77.0)	30.6 (5.2)	NA	NA	NA	NA	NA	NA	NA	NA	NA	NA	NA
Bakris 2014 [29]	https://doi.org/10.1016/j.jacc.2014.05.012	USA	prospective, blinded, randomized, sham-controlled trial.	Patients with resistant hypertension 18 Years to 80 Years (Adult, Older Adult)	Individual is ≥ 18 and ≤ 80 years old at time of randomization. Individual is receiving a stable medication regimen including full tolerated doses of 3 or more anti-hypertensive medications of different classes, of which one must be a diuretic (with no changes for a minimum of 2 weeks prior to screening) that is expected to be maintained without changes for at least 6 months. Individual has an office systolic blood pressure (SBP) of ≥ 160 mmHg based on an average of 3 blood pressure readings measured at both an initial screening visit and a confirmatory screening visit	radiofrequency RDN	sham	The primary efficacy endpoint was a comparison of office SBP change from baseline to 6 months in the renal denervation group compared with the SBP change from baseline to 6 months in the sham control group and required a superiority margin of 5 mm Hg for success. The secondary efficacy endpoint was the change in mean 24-h ambulatory SBP at 6 months. In addition to 24-h ABPM, daytime and nighttime ambulatory BP differences from baseline to 6 months, as well as differences in the change between the 2 groups, were assessed. ABPM differences in BP and heart rate variability	6 months	This trial did not demonstrate a benefit of renal artery denervation on reduction in ambulatory BP in either the 24-h or day and night periods compared with sham (Renal Denervation in Patients With Uncontrolled Hypertension [SYMPLICITY HTN-3]	535	radiofrequency RDN	364	57.9 (10.4)	215 (59.1)	34.2 (6.5)	NA	NA	NA	NA	NA	NA	NA	NA	NA	NA	NA
												sham	171	56.2 (11.2)	110 (64.3)	33.9 (6.4)	NA	NA	NA	NA	NA	NA	NA	NA	NA	NA	NA
Bakris 2015 [34]	https://doi.org/10.1016/j.jacc.2015.01.037	USA	prospective, unblinded, randomized, sham-controlled trial.	Patients with resistant hypertension 18 Years to 80 Years (Adult, Older Adult)	Individual is ≥ 18 and ≤ 80 years old at time of randomization. Individual is receiving a stable medication regimen including full tolerated doses of 3 or more anti-hypertensive medications of different classes, of which one must be a diuretic (with no changes for a minimum of 2 weeks prior to screening) that is expected to be maintained without changes for at least 6 months. Individual has an office systolic blood pressure (SBP) of ≥ 160 mmHg based on an average of 3 blood pressure readings measured at both an initial screening visit and a confirmatory screening visit	radiofrequency RDN	sham	The primary efficacy endpoint was a comparison of office SBP change from baseline to 6 months in the renal denervation group compared with the SBP change from baseline to 6 months in the sham control group and required a superiority margin of 5 mm Hg for success. The secondary efficacy endpoint was the change in mean 24-h ambulatory SBP at 6 months. In addition to 24-h ABPM, daytime and nighttime ambulatory BP differences from baseline to 6 months, as well as differences in the change between the 2 groups, were assessed. ABPM differences in BP and heart rate variability	12 months	These data support no further reduction in office or ambulatory BP after 1-year follow-up. Loss of BP reduction in the non-crossover group may reflect decreased medication adherence or other related factors. (Renal Denervation in Patients With Uncontrolled Hypertension [SYMPLICITY HTN-3]	370	radiofrequency RDN	322	57.9 (10.4)	215/364 (59.1%)	34.2 (6.5)	NA	NA	NA	NA	NA	NA	NA	NA	NA	NA	NA
												sham	48	57.7 (11.8)	47/70 (67.1%)	35.2 (7.8)	NA	NA	NA	NA	NA	NA	NA	NA	NA	NA	NA
Bergland 2021 [35]	https://doi.org/10.1080/08037051.2020.1828818	Norway	Allocation: Randomized Intervention Model: Parallel Assignment Masking: None (Open Label)	Patients with treatment-resistant hypertension, defined as daytime sys- tolic ambulatory BP 135 mmHg after witnessed intake of 3 antihypertensive drugs including a diuretic,	TRH was defined as office systolic BP (SBP) >140 mm Hg, despite maximally tolerated doses of ≥3 antihypertensive drugs including a diuretic. In addition, ambulatory daytime SBP >135 mm Hg after witnessed intake of antihypertensive drugs was required	renal denervation (not mentioned)	Drug Adjustment group	Absolute change in office systolic blood pressure(SBP)	3, 7 years	BP changes up to 7 years show a tendency towards a smaller difference in BPs between the RDN and drug adjustment patients. Our data support RDN as a safe procedure, but it remains non-superior to intensive drug adjustment 7 years after the intervention.	19	RDN	9	57 (10.9)	7(78%)	29 (5.3)	NA	NA	NA	NA	NA	NA	NA	NA	NA	NA	NA
												Drug Adjustment group	10 or 1??	62.7 (5.1)	10 (100%)	30 (5.3)	NA	NA	NA	NA	NA	NA	NA	NA	NA	NA	NA
Bhatt 2014 [18]	DOI: 10.1056/NEJMoa1402670	USA	a prospective, single-blind, randomized, sham-controlled trial.	patients 18 to 80 years of age with resistant hypertension	On initial screening, patients were required to have a systolic blood pressure of 160 mm Hg or higher (average of three measurements at an office visit [hereafter referred to as office blood pressure] while the patient was seated) and to be taking maximally tolerated doses of three or more antihypertensive medications of complementary classes, one of which had to be a diuretic at an appropriate dose. No changes in antihypertensive medication in the previous 2 weeks were allowed. Clinical exclusion criteria were known secondary causes of hypertension and more than one hospitalization for a hypertensive emergency in the previous year. Anatomical exclusion criteria were renal-artery stenosis of more than 50%, renal-artery aneurysm, prior renal-artery intervention, multiple renal arteries, a renal artery of less than 4 mm in diameter, or a treatable segment of less than 20 mm in length.	Radiofrequency	SHAM procedure	Office SBP,24 h SBP	6 m	A total of 535 patients underwent randomization. The mean (±SD) change in systolic blood pressure at 6 months was −14.13±23.93 mm Hg in the denervation group as compared with −11.74±25.94 mm Hg in the sham-procedure group (P<0.001 for both comparisons of the change from baseline), for a difference of −2.39 mm Hg (95% confidence interval [CI], −6.89 to 2.12; P=0.26 for superiority with a margin of 5 mm Hg). The change in 24-hour ambulatory systolic blood pressure was −6.75±15.11 mm Hg in the denervation group and −4.79±17.25 mm Hg in the sham-procedure group, for a difference of −1.96 mm Hg (95% CI, −4.97 to 1.06; P=0.98 for superiority with a margin of 2 mm Hg). There were no significant differences in safety between the two groups.	535	RDN	364	57.9 (10.4)	215 (59.1)	34.2(6.5)	NA	NA	NA	NA	NA	NA	NA	NA	NA	NA	NA
												sham	171	56.2 (11.2)	110 (64.3)	33.9 (6.4)	NA	NA	NA	NA	NA	NA	NA	NA	NA	NA	NA
Böhm 2020 [24]	10.1016/S0140-6736(20)30554-7	44 study sites in Australia, Austria, Canada, Germany, Greece, Ireland, Japan, the UK, and the USA	The SPYRAL Pivotal trial is a multicentre, international, prospective, single-blind, randomised, sham-controlled	Patients were aged 20–80 years at the time of enrolment, had office systolic blood pressure from 150 mm Hg to less than 180 mm Hg and office diastolic blood pressure of at least 90 mm Hg	Patients were aged 20–80 years at the time of enrolment, had office systolic blood pressure from 150 mm Hg to less than 180 mm Hg and office diastolic blood pressure of at least 90 mm Hg. Furthermore, patients were required to have mean 24-h systolic blood pressure of at least 140 mm Hg but less than 170 mm Hg using ambulatory blood pressure monitoring. 24-h blood pressure measurements were considered valid if at least 21 daytime readings and 12 night-time readings had been recorded. Daytime was defined as 0700 h to 2200 h and night-time defined as 2200 h to 0700 h.10 Patients were excluded if they had stable or unstable angina or myocardial infarction within 3 months of enrolment or a history of heart failure, cerebrovascular accident or transient ischaemic attack, or atrial fibrillation. Other key exclusion criteria were renal artery anatomy ineligible for treatment, presence of fibromuscular dysplasia, more than 50% stenosis in any treatable vessel, renal artery stent placed less than 3 months before the procedure, and previous renal denervation. Before randomisation, if patients were on antihypertensive medications, they were required to discontinue them	RF RDN of renal arteries and branches	SHAM procedure	The primary efficacy endpoint was the change in mean 24-h systolic blood pressure from baseline to 3 months after the procedure, adjusted for baseline 24-h systolic blood pressure. The secondary efficacy endpoint was the change in mean office systolic blood pressure from baseline to 3 months after the procedure, adjusted for baseline office systolic blood pressure. Other secondary endpoints were changes in systolic and diastolic blood pressure from baseline at 3, 6, 12, 24, and 36 months; and changes in office systolic and diastolic blood pressure from baseline and incidence of achieving target systolic blood pressure (<140 mm Hg) at 1, 3, 6, 12, 24, and 36 months	3m	s From June 25, 2015, to Oct 15, 2019, 331 patients were randomly assigned to either renal denervation (n=166) or a sham procedure (n=165). The primary and secondary efficacy endpoints were met, with posterior probability of superiority more than 0·999 for both. The treatment difference between the two groups for 24-h systolic blood pressure was –3·9 mm Hg (Bayesian 95% credible interval –6·2 to –1·6) and for office systolic blood pressure the difference was –6·5 mm Hg (–9·6 to –3·5). No major device-related or procedural-related safety events occurred up to 3 months.	331	radiofrequency RDN	166	52·4 (10·9)	107 (64%)	31·1 (6·0)	NA	NA	NA	NA	NA	NA	NA	NA	NA	NA	NA
												SHAM procedure	165	52·6 (10·4)	113 (68%)	30·9 (5·5)	NA	NA	NA	NA	NA	NA	NA	NA	NA	NA	NA
Celinska 2018 [36]	https://doi.org/10.1161/HYPERTENSIONAHA.118.11180	Poland	prospective, phase II, proof-of-concept, randomized, nonsham design, open-label trial	Sixty patients with true resistant hypertension	Inclusion criteria included age between 18 and 70 years, office systolic BP of ≥140 mm Hg (an average of 3 office BP readings), and mean daytime systolic BP on ambulatory BP monitoring (ABPM) of ≥135 mm Hg; receiving and adhering to full doses of ≥3 antihypertensive drug regimen (including a diuretic) for a minimum of 4 weeks before screening; and moderate-to-severe OSA (apnea/hypopnea index [AHI], ≥15 events per hour, with indications for continuous positive airway pressure [CPAP] treatment).The main exclusion criteria included an estimated glomerular filtration rate of ≤60 mL/min, renal artery abnormalities, previous stroke, transient ischemic attack, type 1 diabetes mellitus, or previous implantation of pacemaker or implantable cardioverter defibrillator.	Radiofrequency	control group (maintenance of antihypertensives)	Primary efficacy end point was the difference in mean change in office systolic BP from baseline to 3 months between the RDN group and the control group. The secondary efficacy end points included the difference in mean change in office diastolic BP from baseline to 3 months and systolic and diastolic BP from baseline to 6 months, the difference in mean change in ambulatory systolic and diastolic BP (average from day and night) from baseline to 3 and 6 months, the difference in mean change in AHI from baseline to 3 months, and the difference in mean change in fasting plasma glucose and insulin concentration from baseline to 3 months. The other secondary end points included difference in mean changes in echocardiographic measures at 6 months. The safety end points were mean changes in estimated glomerular filtration rate from baseline to 3 months and incidence of cardiovascular events and arrhythmias from baseline to 3 months as primary safety end point.	3, 6 m		60 patients with true resistant HTN	RDN control group	30 30	55.9±9.4 54.5±9.2	M number=24 (80%) M number=24 (80%)	34.0±6.2 34.7±4.5	NA	NA	NA	NA	NA	NA	NA	NA	NA	NA	NA
Chen 2016 [37]	10.1002/ccd.26594	China	randomized open label trial	age18 and65 years, office systolic BP160 mm Hg, a stabledrug regimen of at least three antihypertensive medications (including a diuretic) with no significant changes for 1 month before enrollment, no known secondary causes of hypertension, and an estimated glomerular filtration rate (eGFR) of45 mL min1/1.73 m^2^ (estimated with the modification of diet in renal disease formula).	The inclusion criteria were as follows: age 18 and 65 years, office systolic BP 160 mm Hg, a stabledrug regimen of at least three antihypertensive medications (including a diuretic) with no significant changes for 1 month before enrollment, no known secondary causes of hypertension, and an estimated glomerular filtration rate (eGFR) of 45 mL min 1/1.73 m^2 ^(estimated with the modification of diet in renal disease formula).	ablation from distal to ostial of bilateral renal arteries	ablation at proximal of bilateral renal arteries	office BP and Ambulatory blood pressure	1,3,6,12m	Compared with full-length ablation, proximal ablation reduced the number of ablation points in both the right (6.1±0.7 vs. 3.3±0.6, P<0.001) and the left renal arteries (6.2±0.7 vs. 3.3±0.8, P<0.001), with significantly shorter RF delivery time (P<0.001), but higher RF power (P=0.011). Baseline office BPs was 179.4±13.7/102.8 9.4 mm Hg in the full-length group and 181.9±12.8/103.5±8.9 mm Hg in the proximal group (P>0.5). Similar office BPs was reduced by −39.4±11.5/−20.9±7.1 mm Hg at 6 months and −38.2±10.3/−21.5±5.8 mm Hg at 12 months in the full-length group (P<0.001), −42.0±11.6/−21.4±7.9 mm Hg at 6 months and −40.9±10.3/−22.1±5.6 mm Hg at 12 months in the proximal group (P<0.001), and progressive BP reductions were observed over the 6 months (P<0.001) in both groups. The drop in ambulatory 24-hr SBP and DBP were significantly less than the drop in office BP (P<0.001). No renovascular or other adverse complications were observed.	47	ablation from distal to ostial of bilateral renal arteries (full length group)	23	47.4±10.13	16 (76.2%)	27.6±2.2	NA	NA	NA	NA	NA	NA	NA	NA	NA	NA	NA
												ablation at proximal of bilateral renal arteries (broximal group)	24	49.8±9.3	14 (73.7%)	26.9±2.4	NA	NA	NA	NA	NA	NA	NA	NA	NA	NA	NA
Desch 2015 [38]	10.1161/HYPERTENSIONAHA.115.05283	Germany	Randomized, single blinde, Sham-Controlled, Trial		Inclusion Criteria: Refractory hypertension: 3 or more antihypertensive agents of different classes (including a diuretic) at optimal dosage without change in the 4 weeks preceding randomization Systolic blood pressure of 135-149 and/or diastolic blood pressure of 90-94 mmHg (ABPM mean daytime values) No change in blood pressure medication within 6 months after randomization Age 18 to 75 years Informed consent	Radiofrequency renal denervation+AHT	sham procedure +AHT	change in 24-hour systolic BP at 6 months between groups in the intention to treat population. Secondary end points included the change in diastolic and mean BP at 6 months and the change in 24-hour mean systolic BP in the per-protocol population. In addition, safety events, such as procedural vascular complications and all-cause death, were analyzed.	6m	A total of 71 patients underwent randomization. Baseline day-time systolic BP was 144.4±4.8 mm Hg in patients assigned to denervation and 143.0±4.7 mm Hg in patients randomized to the sham procedure. The mean change in 24-hour systolic BP in the intention to treat cohort at 6 months was −7.0 mm Hg (95% confidence interval, −10.8 to −3.2) for patients undergoing denervation and −3.5 mm Hg (95% confidence interval, −6.7 to −0.2) in the sham group (P=0.15). In the per protocol population, the change in 24-hour systolic BP at 6 months was −8.3 mm Hg (95% confidence interval, −11.7 to −5.0) for patients undergoing denervation and −3.5 mm Hg (95% confidence interval, −6.8 to −0.2) in the sham group (P=0.042). In patients with mild resistant hypertension, renal sympathetic denervation failed to show a significant reduction in the primary end point of 24-hour systolic BP at 6 months between groups in the intention to treat analysis.	71	Radiofrequency renal denervation+AHT	35	64.5±7.6	27 (77)	31.9±4.4	NA	NA	NA	NA	NA	NA	NA	NA	NA	NA	NA
												sham procedure +AHT	36	57.4±8.6	25 (69)	31.2±4.6	NA	NA	NA	NA	NA	NA	NA	NA	NA	NA	NA
de Jager 2017 (SYMPATHY trial) [39]	10.1161/HYPERTENSIONAHA.116.08818	Netherlands	multicenter, randomized, prospective open label controlled trial	resistant hypertension	Inclusion criteria 1. Individual has a mean daytime SBP ≥135 mm Hg, determined using ABPM, while the patient uses ≥3 antihypertensive agents for ≥3 m before inclusion. 2. Individual is ≥18 years of age	Radiofrequency renal denervation+AHT	AHT	The primary effectiveness end point is change in BP (average ambulatory daytime SBP) after 6 months of follow-up in the intervention group compared with control group	6m	RDN as therapy for resistant hypertension was not superior to usual care. Objective assessment of medication use shows that medication adherence is extremely poor, when patients are unaware of monitoring. Changes over time in adherence are common and affect treatment estimates considerably. Objective measurement of medication adherence during follow-up is strongly recommended in randomized trials	139	Radiofrequency renal denervation+AHT	95	62 (12)	40 (42.1)	28.6 (4.8)	NA	NA	NA	NA	NA	NA	NA	NA	NA	NA	NA
												AHT	44	60 (10)	13 (29.5)	29.4 (4.6)	NA	NA	NA	NA	NA	NA	NA	NA	NA	NA	NA
Elmula 2014 [40]	https://doi.org/10.1161/HYPERTENSIONAHA.114.03246	Norway	open label randomized trial	Patients With True Treatment-Resistant Hypertension	Patients with secondary and spurious hypertension, and some patients with high serum aldosterone levels (primary hyperaldosteronisme without tumor or with high aldosterone/renin activity ratio) who responded to treatment with spironolactone, were identified and excluded. Spironolactone was not given routinely to all patients because this is not considered to be evidence based medicine by European hypertension guidelines. TRH was defined as uncontrolled hypertension (office systolic BP [SBP] >140 mm Hg), despite regular intake of maximally tolerated doses of ≥3 antihypertensive drugs including a diuretic. In addition, patients had to qualify by having mean ambulatory daytime SBP >135 mm Hg immediately after investigator witnessed intake of their antihypertensive morning drugs	Catheter-Based RDN (radiofrequency catheter) + AHT	drug-adjusted group	Absolute change in office systolic blood pressure(SBP)	6m	SBP and diastolic BP were significantly lower in the drug-adjusted group at 6 months (P=0.002 and P=0.004, respectively), and absolute changes in SBP were larger in the drug-adjusted group (P=0.008). Ambulatory BPs changed in parallel to office BPs. Our data suggest that adjusted drug treatment has superior BP lowering effects compared with RDN in patients with true TRH.	19	Catheter-Based RDN (radiofrequency catheter) + AHT	9	57 (10.9)	8 (88.8%)	29 (5.3)	NA	NA	NA	NA	NA	NA	NA	NA	NA	NA	NA
												drug-adjusted group	10	62.7 (5.1)	10 (100%)	30 (5.3)	NA	NA	NA	NA	NA	NA	NA	NA	NA	NA	NA
Engholm 2018 [41]	10.1016/j.ijcard.2017.09.200	Denmark	doubleblinded, randomized, sham-controlled trial	patients with treatment-resistant hypertension	Patients eligible for inclusion were aged 30-70 years, with a systolic daytime ABPM of 145 mmHg or more, despite stable treatment with three or more antihypertensive drugs including a diuretic when tolerated. ABPM were preceded by 14 days of scheduled drug intake and drug compliance registration, demonstrating minimum 85% adherence	Catheter-Based RDN (radiofrequency catheter) + AHT	sham procedure	Coronary flow reserve (CFR) and coronary- and forearm minimum vascular resistance (C-Rmin and F-Rmin), ASBP	6m	RDN was performed with 5.3±0.2 lesions in the right renal artery and 5.4±0.2 lesions in the left. Baseline ASBP was 152±2 mmHg (RDN, n=29) and 154±2 mmHg (SHAM, n=29). Similar reductions in MAP were seen at follow up (-3.5±2.0 vs. -3.2±1.8, p=0.92). Baseline CFR was 2.9±0.1 (RDN) and 2.4±0.1 (SHAM), with no significant change at follow-up (0.2±0.2 vs. -0.1±0.2, P=0.57). C-Rmin was 1.9±0.3 (RDN) and 2.7±0.6 (SHAM) (mmHg min/ml pr. 100 g) and did not change significantly (0.3±0.5 vs. -0.4±0.8, P=0.48). F-Rmin was 3.6±0.2 (RDN) and 3.6±0.3 (SHAM) (mmHg min/ml pr. 100 ml tissue) and unchanged at follow-up (4.2±0.4 vs. 3.8±0.2, P=0.17). Left ventricular mass index was unchanged following RDN (-4±7 (RDN) vs. 3±5 (SHAM) (g/m^2^) P=0.38).	58	Catheter-Based RDN (radiofrequency catheter) + AHT	29	54.1±1.4	21 (72)	27.9±0.9	NA	NA	NA	NA	NA	NA	NA	NA	NA	NA	NA
												sham procedure	29	57.7±1.8	21 (72)	28.6±0.7	NA	NA	NA	NA	NA	NA	NA	NA	NA	NA	NA
Esler 2010 [17]	https://doi.org/10.1016/s0140-6736(10)62039-9	Europe, Australia, and New Zealand	multicentre, prospective, randomised trial	patients with treatment-resistant hypertension	Patients aged 18–85 years with a systolic blood pressure of 160 mm Hg or more (≥150 mm Hg in patients with type 2 diabetes), despite compliance with three or more antihypertensive drugs, were eligible for inclusion. Exclusion criteria included an estimated glomerular filtration rate (eGFR; based on the Modification of Diet in Renal Disease criteria 12) of less than 45 mL/min per 1·73 m², type 1 diabetes, contraindications to MRI, substantial stenotic valvular heart disease, pregnancy or planned pregnancy during the study, and a history of myocardial infarction, unstable angina, or cerebrovascular accident in the previous 6 months	Radiofrequency	AHT	The primary effectiveness endpoint was between-group change in average office-based measurements of systolic blood pressure from baseline to 6 months after randomisation	6m	106 (56%) of 190 patients screened for eligibility were randomly allocated to renal denervation (n=52) or control (n=54) groups between June 9, 2009, and Jan 15, 2010. 49 (94%) of 52 patients who underwent renal denervation and 51 (94%) of 54 controls were assessed for the primary endpoint at 6 months. Office-based blood pressure measurements in the renal denervation group reduced by 32/12 mm Hg (SD 23/11, baseline of 178/96 mm Hg, p<0·0001), whereas they did not differ from baseline in the control group (change of 1/0 mm Hg [21/10], baseline of 178/97 mm Hg, p=0·77 systolic and p=0·83 diastolic). Between-group differences in blood pressure at 6 months were 33/11 mm Hg (p<0·0001). At 6 months, 41 (84%) of 49 patients who underwent renal denervation had a reduction in systolic blood pressure of 10 mm Hg or more, compared with 18 (35%) of 51 controls (p<0·0001). We noted no serious procedure-related or device-related complications and occurrence of adverse events did not diff er between groups; one patient who had renal denervation had possible progression of an underlying atherosclerotic lesion, but required no treatment	106	Radiofrequency	52	58 (12)	34(65)	31 (5)	NA	NA	NA	NA	NA	NA	NA	NA	NA	NA	NA
												AHT	54	58 (12)	27(50)	31 (5)	NA	NA	NA	NA	NA	NA	NA	NA	NA	NA	NA
Fengler 2019 [42]	https://doi.org/10.1161/CIRCULATIONAHA.118.037654	Germany	single-blind, single-center, 3-arm randomized clinical trial	patients with resistant hypertension	Patients were screened if diagnosed with resistant hypertension (office BP >160 mmHg systolic or >90 mmHg diastolic, despite treatment with ≥3 different classes of antihyper- tensive drugs on at least 50% of maximum dosage for hypertension, including at least 1 diuretic unless intolerant to diuretics). Antihypertensive medication had to be stable for at least 4 weeks. Patients then underwent ambulatory blood pressure measurement (ABPM) to exclude white-coat hyper- tension. Inclusion criteria were resistant hypertension with systolic daytime BP >135 mmHg on ABPM and renal artery diameter of at least 1 main renal artery ≥5.5 mm.	radiofrequency ablation of the main renal artery (RFM-RDN) versus radiofrequency ablation of the main renal artery, branches, and accessories (RFB-RDN)	ultrasound-based ablation of the main renal artery (USM-RDN)	24-h systolic BP, 24-h diastolic BP,Day-time systolic BP, Day-time diastolic BP, Night-time systolic BP, Night-time diastolic BP,	3 months	blood pressure was significantly more reduced in the ultrasound ablation group than in the radiofrequency ablation group of the main renal artery. No significant difference was found between the radiofrequency ablation groups for additional side branch ablation. USM-RDN is superior over RFM-RDN, whereas RFB-RDN was not superior to RFM-RDN.	120	radiofrequency ablation of the main renal artery (RFM-RDN)	39	63.8±9.9	male 26 (67%)	30.6±5.4	NA	NA	NA	NA	NA	NA	NA	NA	NA	NA	NA
												radiofrequency ablation of the main renal artery, branches, and accessories (RFB-RDN)	39	62.1±10.2	male 24 (62%)	31.6±5.9	NA	NA	NA	NA	NA	NA	NA	NA	NA	NA	NA
												ultrasound-based ablation of the main renal artery (USM-RDN)	42	64.6±8.0	32 (76%)	32.6±5.4	NA	NA	NA	NA	NA	NA	NA	NA	NA	NA	NA
Gao 2021 [43]	http://doi.org/10.15212/CVIA.2021.0023	China	single-center, prospective, randomized, single-blind (patient-blind) clinical trial	patients with resistant hypertension	Male or female, aged 18–85 years. patients have been receiving three or more antihypertensive drugs for at least 4 weeks. Blood pressure of 140/90 mmHg or higher at the time of screening	Microtube-irrigated ablation catheter	General ablation catheter	24 h SBP, 24 h DBP, Heart rate, LVESd, LVPW thickness, ejection fraction, interventricular septal thickness, LVEDd, left ventricular end-diastolic diameter, left ventricular end-systolic diameter, left ventricular posterior wall, systolic blood pressure, serum creatinine.	6, 12 months	At the 6-month follow-up, ambulatory blood pressure in the microtube-irrigated ablation catheter group was significantly lower than in the general ablation catheter group. At the 12-month follow-up, the between-group difference in ambulatory blood pressure was not statistically significant. At the 12-month follow-up, the number of antihypertensive drugs and diuretics used in the microtube-irrigated ablation catheter group was less than in the general ablation catheter group	60	Microtube-irrigated ablation catheter	30	55.93±10.94	8 (26.7%)	NA	NA	NA	NA	NA	NA	NA	NA	NA	NA	NA	NA
												General ablation catheter	30	62.73±12.50	8 (26.7%)	NA	NA	NA	NA	NA	NA	NA	NA	NA	NA	NA	NA
Gosse 2017 [44]	10.1161/HYPERTENSIONAHA.116.08448	France	RCT	A total of 44 out of 53 patients randomized to the RD group and 53 out of 53 randomized to the control group were included in the per-protocol analysis. Nine patients randomized to the RD group were not included for the following reasons: 3 refused the procedure after inclusion, 3 had ineligible renal artery anatomy diagnosed during the procedure, 1 had a complication before RD, and 2 had missing ABPM data at 6 months		Radiofrequency	SSAHT	24h SBP,DBP Day SBP.DBP night SBP,DBP	6m		97	RDN	44			30.80±5.09	NA	NA	NA	NA	NA	NA	NA	NA	NA	NA	NA
												SSAHT	53			29.73±4.54	NA	NA	NA	NA	NA	NA	NA	NA	NA	NA	NA
Hering 2013 [45]	https://doi.org/10.1161/HYPERTENSIONAHA.111.00194	Australia	prospective controlled trial	patients with resistant hypertension	patients without evidence of secondary forms of hypertension such as primary aldosteronism, renovascular hypertension, pheochromocytoma, Cushing’s disease, and others as assessed by physical examination, biochemical and imaging studies.	Catheter-Based RDN (radiofrequency catheter) + AHT	usual medical care	office systolic blood pressure, office diastolic blood pressure, heart rate, muscle sympathetic nerve activity.	3 months	Mean office BP decreased significantly with RDN but not in non-RDN at 3-month follow-up. RDN moderately decreased multi-unit muscle sympathetic nerve activity.	35	Catheter-Based RDN (radiofrequency catheter) + AHT	25	57±2	72% male	NA	NA	NA	NA	NA	NA	NA	NA	NA	NA	NA	NA
												usual medical care	10	59±4	90%	NA	NA	NA	NA	NA	NA	NA	NA	NA	NA	NA	NA
Jacobs 2017 [46]	http://dx.doi.org/10.1080/08037051.2017.1320939	The Netherlands	multicenter randomized controlled trial	patients with resistant hypertension	Eligible patients have essential hypertension, are younger than 70 years, and have a glomerular filtration rate estimated from serum creatinine by the "Chronic Kidney Disease Epidemiology Collaboration (CKD-EPI) equation" not less than 60 mL/min/m^2^ at screening and per amendment to the trial protocol (No. B3222014197090; 23 December 2014) not less than 45 mL/min/m^2^ at the last run-in visit.	anti-hypertensive drug plus RDN	usual medical treatment " anti-hypertensive drug"	office blood pressure, 24-hours blood pressure, day-time BP, night-time BP, differences in ECG measurements.	6 months	Six months after randomization, the 24-h and nighttime ambulatory blood pressure decreased significantly more in response to RDN on top of optimized antihypertensive drug treatment compared with sole optimization of drug treatment	15	anti-hypertensive drug plus RDN	6	48.4±10.8	3 (50%)`	29.3±4.5	NA	NA	NA	NA	NA	NA	NA	NA	NA	NA	NA
												usual medical treatment " anti-hypertensive drug"	9	47.9±8.8	4 (44.4%)	31.0±4.9	NA	NA	NA	NA	NA	NA	NA	NA	NA	NA	NA
Kandzari 2018 [20]	https://doi.org/10.1016/S0140-6736(18)30951-6	USA, Germany, Japan, UK, Australia, Austria, and Greece.	s international, randomised, single-blind, sham-control	patients with uncontrolled hypertension (aged 20–80 years)	patients who aged 20–80 years and had an office systolic blood pressure between 150 and 180 mm Hg, an office diastolic blood pressure of 90 mm Hg or higher, and a mean 24 h ambulatory systolic blood pressure between 140 and 170 mm Hg. Patients were on one, two, or three standard antihypertensive drugs.	radiofrequency RDN ablation treatments in a spiral pattern in the four quadrants of the renal artery and branch vessels between three and eight mm in diameter	Sham procedure	24h SBP,DBP Day SBP.DBP night SBP,DBP	6 months	Office and 24 h ambulatory blood pressure decreased significantly from baseline to 6 months in the renal denervation group. The change in blood pressure was significantly greater at 6 months in the renal denervation group than the sham-control group.	80	radiofrequency RDN ablation treatments in a spiral pattern in the four quadrants of the renal artery and branch vessels between three and eight mm in diameter	38	53·9 (8·7)	33 (87%)	31·4 (6·4)	NA	NA	NA	NA	NA	NA	NA	NA	NA	NA	NA
												Sham procedure	42	53·0 (10·7)	34 (81%)	32·5 (4·6)	NA	NA	NA	NA	NA	NA	NA	NA	NA	NA	NA
Kario 2015 [47]	10.1253/circj.CJ-15-0150	Japan	a prospective, randomized, controlled clinical trial	patients with resistant hypertension	subjects who were at least 20 years old and ≤80 years old at the time of informed consent. Subjects were required to have uncontrolled hypertension defined as office SBP ≥160mmHg while on a stable anti-hypertensive regimen of at least 3 anti-hypertensive drug classes at maximum tolerated dose including a diuretic for a minimum of 6 weeks prior to enrollment; 24-h average ambulatory SBP was required to be ≥135mmHg.	catheter based renal denervation	sham control	24-h ambulatory SBP, office SBP, adverse events	6 months	There were no significant reductions in SBP by any measure in the control subjects. The difference in office SBP change between the groups, however, was not significantly different and thus the primary effectiveness endpoint was not met.	41	catheter based renal denervation	22	59.5±11.9	15 (68.2%)	27.0±5.5	NA	NA	NA	NA	NA	NA	NA	NA	NA	NA	NA
												sham control	19	56.0±13.0	16 (84.2%)	28.0±3.9	NA	NA	NA	NA	NA	NA	NA	NA	NA	NA	NA
Kario2022B [48]	10.1038/s41440-022-01042-8	International	The SPYRAL HTN-ON MED trial is an international, prospective, randomized, blinded, sham-controlled trial	resistant hypertension patients taking 1-3 hypertension medications	eligible patients were aged 20–75 years and had resistant hypertension (average seated office BP≥ 150/90mmHg) despite treatment with a stable regimen including maximum tolerated dosages of at least three antihypertensive medications from different classes (including a diuretic) and 24-hour ambulatory systolic BP (SBP) of ≥140mmHg during a screening period of ~4–8 weeks prior to the procedure. Renal artery anatomy eligibility was determined using computed tomography or magnetic resonance angiogram at the end of the screening period, then confirmed by renal artery angiography at the time of procedure. Patients with unsuitable renal artery anatomy were excluded, as were those with chronic kidney disease (estimated glomerular filtration rate <40 mL/min/1.73 m^2^), secondary hypertension (although patients with sleep apnea were eligible), inadequately controlled diabetes mellitus, inflammatory bowel disease, history of severe cardiovascular event, or other chronic conditions. Briefly, patients were enrolled with office systolic BP (SBP)≥150mmHg and <180mmHg, office diastolic BP (DBP)≥90mmHg, mean 24h SBP≥140mmHg and <170mmHg, and prescribed 1–3 antihypertensive medications at baseline.	RF ablation of main renal artery, accessory and branch vessels	sham control	Long-term safety was compared between RDN and sham control groups through 36 months using a composite endpoint of major adverse events. Long-term efficacy was evaluated from baseline to 36 months. Nighttime (1:00–6:00 AM) and morning (7:00–9:00 AM) BPs were separately calculated from the average of BPs. Morning BP parameters were defined as follows: average morning SBP (average of morning SBPs), moving peak morning SBP (highest 1h moving average of 3 consecutive SBPs over the morning period) and minimum (lowest recorded value) and maximum (highest recorded value during the morning interval) morning SBP. Nighttime BP parameters were similarly defined as follows: average nighttime SBP (average of nighttime interval SBPs), average peak nighttime SBP (average of the 3 highest measurements over the nighttime interval) and minimum (lowest recorded value) and maximum (highest recorded value during the nighttime interval) nighttime SBP.	36m	Morning and nighttime SBP were significantly lower in patients prescribed at least 3 antihypertensive medications at 36 months in the SPYRAL HTN-ON MED trial for RDN compared with sham control. The results suggest RDN has significant benefit when the risk of cardiovascular events is highest.	46	RDN	23	55.1±8.8	22 (95.7%)	32.3±5.4	NA	NA	NA	NA	NA	NA	NA	NA	NA	NA	NA
												sham	23	51.0±10.2	19 (82.6%)	33.8±4.5	NA	NA	NA	NA	NA	NA	NA	NA	NA	NA	NA
Kario2022A [49]	10.1038/s41440-021-00754-7	Japan and South Korea	a multicenter randomized, single-blind, sham-controlled trial	resistant hypertension	eligible patients were aged 20–75 years and had resistant hypertension (average seated office BP ≥ 150/90 mmHg) despite treatment with a stable regimen including maximum tolerated dosages of at least three antihypertensive medications from different classes (including a diuretic) and 24-hour ambulatory systolic BP (SBP) of ≥140 mmHg during a screening period of ~4–8 weeks prior to the procedure. Renal artery anatomy eligibility was determined using computed tomography or magnetic resonance angiogram at the end of the screening period, then confirmed by renal artery angiography at the time of procedure. Patients with unsuitable renal artery anatomy were excluded, as were those with chronic kidney disease (estimated glomerular filtration rate <40 mL/min/1.73 m^2^), secondary hypertension (although patients with sleep apnea were eligible), inadequately controlled diabetes mellitus, inflammatory bowel disease, history of severe cardiovascular event, or other chronic conditions.	bilateral US main renal artery denervation	sham control	The primary endpoint was the between-group difference in change in 24-hour ambulatory SBP from baseline at 3 months. Secondary endpoints were change in daytime and nighttime ambulatory SBP from baseline at 3 months, change in 24-hour, daytime and nighttime ambulatory diastolic BP (DBP) from baseline at 3 months, and change in seated office SBP and DBP from baseline at 3 months. Other prespecified observational endpoints include change in home SBP and DBP	3 m	This study did not show a significant difference in ambulatory blood pressure reductions between renal denervation and a sham procedure in treated patients with resistant hypertension. Although blood pressure reduction after renal denervation was similar to other sham-controlled studies, the sham group in this study showed much greater reduction. This unexpected blood pressure reduction in the sham control group highlights study design issues that will be addressed in a new trial.	143	RDN	69	50.7±11.4	48 (69.6%)	29.5±5.5	NA	NA	NA	NA	NA	NA	NA	NA	NA	NA	NA
												Sham	67	55.6±12.1	53 (79.1%)	28.4±4.5	NA	NA	NA	NA	NA	NA	NA	NA	NA	NA	NA
Mahfoud 2022 [50]	10.1016/S0140-6736(22)00455-X	International: USA, Germany, Japan, the UK, Australia, Austria, and Greece,	s randomised, single-blind, sham-controlled trial	resistant hypertension taking at least 3 medications	if they had office systolic blood pressure of at least 150 mm Hg but less than 180 mm Hg, office diastolic blood pressure of at least 90 mm Hg, mean 24-h systolic blood pressure of at least 140 mm Hg but less than 170 mm Hg, and were taking one to three antihypertensive medications	RF RDN of renal arteries and branches	sham control	The primary endpoint was the treatment difference in mean 24-h systolic blood pressure at 6 months between the renal denervation group and the sham control group	24, 36 m	Radiofrequency renal denervation compared with sham control produced a clinically meaningful and lasting blood pressure reduction up to 36 months of follow-up	80	RDN	38	53·9 (8·7)	33 (87%)	31·4 (6·4)	NA	NA	NA	NA	NA	NA	NA	NA	NA	NA	NA
												Sham	42	53·0 (10·7)	34 (81%)	32·5 (4·6)	NA	NA	NA	NA	NA	NA	NA	NA	NA	NA	NA
Mathiassen 2016 [51]	https://doi.org/10.1097%2FHJH.0000000000000977	Denmark	RCT	Patients with resistant hypertension	Age (30–70) years with One month of stable antihypertensive treatment with at least three antihypertensive agents including a diuretic (or in case of diuretics intolerance a minimum of three nondiuretic antihypertensive drugs), Daytime ABPM SBP >=145 mmHg (preceded by 14 days of scheduled drug intake showing at least 85% adherence)	radiofrequency renal denervation	sham control	day time ambulatory blood pressure monitoring, Mean usage of antihypertensive medication, adverse events, and effect on vasoactive hormones	1, 3, 6 months	Both Groups had similar reductions in daytime systolic ABPM compared with baseline at 3 months [6.21(8.8) mmHg in RDN group vs. 6.0 (13.5) mmHg in SHAM group] and at 6 months [6.1 (18.9) mmHg in RDN group vs. 4.3 (15.1) mmHg in SHAM].Mean change in antihypertensive medication usage at 3 months was equal between both groups.	69	radiofrequency renal denervation	36	54.3 (7.8)	75%	28.2 (5.0)	NA	NA	NA	NA	NA	NA	NA	NA	NA	NA	NA
												SHAM	33	57.1 (9.6)	73%	28.8 (3.9)	NA	NA	NA	NA	NA	NA	NA	NA	NA	NA	NA
Oliveras 2016 [52]	10.1097/HJH.0000000000001025	Spain	prospective, multicentre, open-label, randomized, controlled trial	patients enrolled from October 2012 to April 2015 at three tertiary care centres specialized for hypertension diagnosis and management, all in Catalonia, Spain.	Patients aged at least 18 years and 80 years or less with an office SBP at least 150 mmHg and a 24-h SBP at least 140 mmHg despite a prescribed therapeutic schedule with an appropriate combination of three or more full-dose antihypertensive drugs, including a diuretic, and maintained for the last 3 months,	Radiofrequency in main renal artery	spironolactone	Mean changes in ambulatory 24-h SBP from baseline to 6 months, mean changes in BP and heart rate parameters from baseline to 6 months AND adverse events	6 months	Spironolactone was more effective than RDN in reducing 24-h SBP and 24-h DBP. The decrease in estimated glomerular filtration rate was greater in the spironolactone group compared with RDN group.	24	RDN	11	61.9 (6.6)	55%	33.7 (7.4)	NA	NA	NA	NA	NA	NA	NA	NA	NA	NA	NA
												Spironolactone	13	64.9 (8.2)	69%	30.6 (3.6)	NA	NA	NA	NA	NA	NA	NA	NA	NA	NA	NA
Pekarskiy 2017 [53]	10.1097/HJH.0000000000001160	Russia	single-center, double-blind, randomized, controlled, and parallel group study	The study population included patients with uncontrolled hypertension consecutively referred to the Tomsk Research Institute of Cardiology for extended evaluation by the hypertension specialist to confirm the diagnosis of true resistant hypertension. Eligible patients included men and women 18–80 years of age with office SBP at least 160 mmHg or DBP at least 100 mmHg despite stable (>3 months) prescribed treatment with full doses of at least three antihypertensive drugs, including a diuretic.	Patients were excluded if they had secondary hypertension, 24-h mean SBP less than 135 mmHg, estimated glomerular filtration rate (eGFR) [by Modification of Diet in Renal Disease (MDRD) study formula] less than 30 ml/min per 1.73 m^2^, extended disease of the renal arteries, or any other severe comorbidity significantly increasing risk of the intervention (investigator’s assessment)	RFA of Main Renal Artery	RFA of Renal Artery branches	24-h mean ambulatory SBP	6 months	. Six months after randomization, the distal therapy group (n¼ 24) had a significantly greater decrease in the primary outcome, 24-h mean ambulatory SBP, as compared with the conventionally treated group	51	RFA of Main Renal Artery (conventional)	26	56.7 (9.1)	10 (39%)	-	NA	NA	NA	NA	NA	NA	NA	NA	NA	NA	NA
												RFA of Renal Artery branches (distal)	25	54.7 (8.2)	10 (40%)	-	NA	NA	NA	NA	NA	NA	NA	NA	NA	NA	NA
Peters 2017 [54]	10.1080/08037051.2017.1368368	Denmark	randomized, sham-controlled, double-blinded	patients with resistant hypertension who came to outpatient clinics, in the Central Region of Denmark.	Age [30–70] years; 1 month of stable treatment with at least 3 antihypertensive agents including a diuretic (or in case of diuretics intolerance a minimum of 3 non-diuretic drugs), daytime ABPM systolic blood pressure 145 mmHg, preceded by 14 days of scheduled drug intake showing at least 85% adherence	radiofrequency RDN single point of ablation	SHAM procedure	systolic 24-hour ambulatory BP, change in pulse wave velocity, 24h systolic ABPM, 24h diastolic ABPM, heart rate variability (HRV), arterial stiffness, central blood pressure (C-BP)	6 months	no significant effects of RDN on arterial stiffness, C-BP and HRV. There was no significant effect of RDN on peripheral brachial BP and 24-h ABPM in a sham-controlled setting	53	radiofrequency RDN single point of ablation	26	54±8	65%	28±5	NA	NA	NA	NA	NA	NA	NA	NA	NA	NA	NA
												SHAM procedure	27	59±9	78%	30±3	NA	NA	NA	NA	NA	NA	NA	NA	NA	NA	NA
Rosa 2015 [55]	10.1161/HYPERTENSIONAHA.114.04019	Czech Republic	prospective, randomized, open-label multicenter trial	True-Resistant Hypertension patients	patients with RH were included (with an office systolic BP >140 mm Hg after treatment with ≥3 antihypertensive drugs at optimal doses, including a diuretic). Secondary causes of hypertension (eg, primary aldosteronism, pheochromocytoma, Cushing syndrome, renal parenchymal disease, renovascular hypertension, drug-induced hypertension, or other conditions) were excluded in all subjects before randomization.	RF bilateral renal denervation plus optimal anti-hypertensive treatment group	intensified pharmacotherapy	office and 24 BP	6 months	renal denervation achieved reduction of blood pressure comparable with intensified pharmacotherapy.	106	RDN+ anti-hypertensive drug	52	56±12	40 (77%)	31.2±4.3	NA	NA	NA	NA	NA	NA	NA	NA	NA	NA	NA
												intensified medical ((PHAR),	54	59±9	34 (63%)	33.4±4.7	NA	NA	NA	NA	NA	NA	NA	NA	NA	NA	NA
Saxena 2018 [56]	DOI: 10.1161/JAHA.118.009151	United Kingdom	single-center Phase II Randomized Sham Controlled Study	subjects with resistant hypertension.	Subjects between 18 and 80 years of age with uncontrolled primary arterial hypertension, defined as office SBP ≥160 mm Hg while taking 3 or more antihypertensive medications at the maximally tolerated doses, one of which was a diuretic, were enrolled in the study. the 24-hour ambulatory BP monitoring average SBP of ≥135 mm Hg and no medication changes within the month before baseline.	bilateral renal denervation using therapeutic levels of ultrasound energy	sham application of diagnostic ultrasound	24h SBP,DBP Day SBP.DBP night SBP,DBP	6 months	There was significant office blood pressure reduction in both treatment and sham groups because of which the study was discontinued prematurely. However, during the late phase II of Valsalva maneuver, renal denervation resulted in substantial and significant reduction in mean arterial pressure with no significant changes in the sham group.	23	bilateral renal denervation using therapeutic levels of ultrasound energy	12	57.2 (10.3)	75%	31.7 (3.4)	NA	NA	NA	NA	NA	NA	NA	NA	NA	NA	NA
												sham application of diagnostic ultrasound	11	61.9 (10.6)	60%	29.7 (4.8)	NA	NA	NA	NA	NA	NA	NA	NA	NA	NA	NA
Schmieder 2017 [57]	DOI: 10.1097/HJH.0000000000001584	Czech Republic, Germany, New Zealand, Poland and the UK	international, randomized, sham-controlled, double-blind study	patients with uncontrolled primary arterial hypertension	Patients between 18 and 80 years of age with uncontrolled primary arterial hypertension, defined as office SBP at least 160 mmHg while taking three or more antihypertensive medications at the maximally tolerated doses, with one required to be a diuretic and 24-h ABPM average SBP of at least 135 mmHg and no medication changes within the month prior to baseline.	bilateral RDN using therapeutic levels of ultrasound energy	sham consisted of bilateral application of diagnostic levels of ultrasound energy	office SBP, 24-h ambulatory SBP, adverse events	3,6 months	there is a similar significant neither changes in office BP at 12 and 24 weeks, and in 24-h ambulatory BP at 24-week follow-up visit between the two groups significantly. No serious adverse events were observed in both groups.	81	bilateral RDN using therapeutic levels of ultrasound energy	42	60.3 (11.2)	male (81.4%)	29.9 (4.5)	NA	NA	NA	NA	NA	NA	NA	NA	NA	NA	NA
												sham consisted of bilateral application of diagnostic levels of ultrasound energy	39	62 (11.1)	male (64.1%)	29.8 (4.2)	NA	NA	NA	NA	NA	NA	NA	NA	NA	NA	NA
Townsend 2017 [21]	10.1016/S0140-6736(17)32281-X	USA, Germany, Japan, UK, Australia, Austria, and Greece	multicentre, international, single-blind, randomised, sham-controlled, proof-of-concept trial	patients with uncontrolled hypertension in the absence of antihypertensive medications	we enrolled patients aged 20–80 years with mild to moderate hypertension (defined as office systolic blood pressure [SBP] ≥150 mm Hg and <180 mm Hg, office diastolic blood pressure [DBP] ≥90 mm Hg, and a mean 24-h ambulatory SBP ≥140 mm Hg and <170 mm Hg).	RF RDN	sham control	office and 24 hour BP	3 months	. Office and 24-h ambulatory blood pressure decreased significantly from baseline to 3 months in the renal denervation group	80	RDN	38	55·8 (10·1)	26 (68·4%)	29·8 (5·1)	NA	NA	NA	NA	NA	NA	NA	NA	NA	NA	NA
												sham	42	52·8 (11·5)	31 (73·8%)	30·2 (5·1)	NA	NA	NA	NA	NA	NA	NA	NA	NA	NA	NA
																	NA	NA	NA	NA	NA	NA	NA	NA	NA	NA	NA
Weber 2020 [58]	10.1016/j.jcin.2019.10.061	USA	randomized, sham-controlled multicenter trial	Eligible patients were 18 to 75 years of age, had office-based systolic BP (SBP) of $150 and #180 mm Hg, and had an average 24-h ambulatory SBP of $135 and #170 mm Hg after 4-week antihypertensive medication washout	• Age ≥18 and ≤75 years • Office Systolic blood pressure (BP) ≥150 mmHg and ≤180 mmHg based on an average of 3 office-based BP measurements • Average 24-hour Ambulatory Systolic BP ≥135 mmHg and ≤170 mmHg • For each kidney, a main renal artery, with or without accessory renal arteries, with diameter ≥3.0 mm and ≤7.0 mm and length ≥20.0 mm • Agrees to have all study procedures performed, and is competent and willing to provide written, informed consent Exclusion Criteria • Previous renal artery intervention or clinically significant renal artery abnormalities that would interfere with safe cannulation of the renal artery • Stenosis >30% or renal artery aneurysm in either renal artery • Fibromuscular dysplasia (FMD) • Platelet count of <90,000 or • >500,000 per microliter (μL) of blood or history of bleeding diathesis • Known causes of secondary hypertension • Type 1 diabetes mellitus • eGFR <40 mL/min/1.73m^2^, as calculated using the MDRD (Modification of Diet in Renal Disease) methodology • Only one kidney or prior transplantation • Contraindicated for anticoagulant therapy and other recommended study medications • Known hypersensitivity or contraindication to contrast dye that, in the opinion of the investigator, cannot be adequately pre-medicated • Pregnant or planning to become pregnant • History of myocardial infarction, unstable angina pectoris, hypertensive crisis, or a cerebrovascular accident in the previous 3 months • Known ejection fraction of <30% or heart failure that required hospitalization in the previous 6 months • Scheduled for or has planned surgery or cardiovascular intervention in the next 6 months • ≥1 episode(s) of orthostatic hypotension not related to medication changes, coupled with symptoms, within the past year or identified during screening • Severe valvular heart disease • History of any serious medical condition, which in the opinion of the investigator, may adversely affect the safety of the participant and/or the scientific integrity of the study • Active implantable device [e.g., implantable cardioverter-defibrillator (ICD), cardiac resynchronization therapy defibrillator (CRT-D), spinal cord stimulators, cochlear implants] • Current participation in another drug or device trial	Radiofrequency	sham-procedure control (renal angiography alone	SBP 24 h DBP 24 h SBP office DBP office	2,6,12 m	Baseline 24-h blood pressure was 148.3 10.9/85.7 9.1 mm Hg for the denervation group (n ¼ 34, mean age 58.5 10.1 years, 47% women) and 149.1 7.2/86.4 9.8 mm Hg for the control group (n ¼ 17, mean age 58.2 9.8 years, 24% women). At 8 weeks, mean 24-h SBP reductions for the renal denervation and control groups were 5.3 mm Hg (95% confidence interval [CI]: 8.8 to 1.8 mm Hg) and 8.5 mm Hg (95% CI: 13.3 to 3.8 mm Hg), respectively (difference 3.3 mm Hg; 95% CI: 2.8 to 9.3 mm Hg; p ¼ 0.30). Antihypertensive medications could then be added. By 6 months, decreases in SBP were greater for the denervation group, yielding between-group differences of 7.2 mm Hg (95% CI: 15.2 to 0.8 mm Hg; p ¼ 0.08), 9.7 mm Hg (95% CI: 17.7 to 1.7 mm Hg; p ¼ 0.02), and 11.4 mm Hg (95% CI: 19.2 to 3.7 mm Hg; p<0.01) for 24-h, daytime ambulatory, and office measurements, respectively. Through 12 months, 1 patient (renal denervation group) had a hypertensive urgency requiring immediate management, and 1 experienced progression of renal artery stenosis.	51	RDN sham procedure	34 17	58.5±10.1 58.2 9.8	18		NA	NA	NA	NA	NA	NA	NA	NA	NA	NA	NA
												Sham procedure	17	58.2±9.8	13		NA	NA	NA	NA	NA	NA	NA	NA	NA	NA	NA
																	NA	NA	NA	NA	NA	NA	NA	NA	NA	NA	NA
											4498						NA	NA	NA	NA	NA	NA	NA	NA	NA	NA	NA

Supplemental material 2

Supplemental material 3

Supplemental material 4

Supplemental material 5

Supplemental material 6

Supplemental material 7

Supplemental material 8

Supplemental material 9

Supplemental material 10

Supplemental material 11

Supplemental material 12

Supplemental material 13

## References

[1] Sarafidis PA, Georgianos P, Bakris GL (2013). Resistant hypertension - its identification and epidemiology. Nat Rev Nephrol.

[2] Egan BM, Zhao Y, Axon RN (2010). US trends in prevalence, awareness, treatment, and control of hypertension, 1988-2008. JAMA.

[3] Kearney PM, Whelton M, Reynolds K, Muntner P, Whelton PK, He J (2005). Global burden of hypertension: analysis of worldwide data. Lancet.

[4] Calhoun DA, Jones D, Textor S (2008). Resistant hypertension: diagnosis, evaluation, and treatment: a scientific statement from the American Heart Association Professional Education Committee of the Council for High Blood pressure research. Circulation.

[5] Davis MI, Filion KB, Zhang D, Eisenberg MJ, Afilalo J, Schiffrin EL, Joyal D (2013). Effectiveness of renal denervation therapy for resistant hypertension: a systematic review and meta-analysis. J Am Coll Cardiol.

[6] Lewington S, Clarke R, Qizilbash N, Peto R, Collins R (2002). Prospective studies collaboration: age-specific relevance of usual blood pressure to vascular mortality: a meta-analysis of individual data for one million adults in 61 prospective studies. Lancet.

[7] Pimenta E, Calhoun DA (2012). Resistant hypertension: incidence, prevalence, and prognosis. Circulation.

[8] Zhou D, Xi B, Zhao M, Wang L, Veeranki SP (2018). Uncontrolled hypertension increases risk of all-cause and cardiovascular disease mortality in US adults: the NHANES III Linked Mortality Study. Sci Rep.

[9] Bakris G, Nathan S (2014). Renal denervation and left ventricular mass regression: a benefit beyond blood pressure reduction?. J Am Coll Cardiol.

[10] Evelyn KA, Singh MM, Chapman WP, Perera GA, Thaler H (1960). Effect of thoracolumbar sympathectomy on the clinical course of primary (essential) hypertension. A ten-year study of 100 sympathectomized patients compared with individually matched, symptomatically treated control subjects. Am J Med.

[11] Hoobler SW, Manning JT, Paine WG (1951). The effects of splanchnicectomy on the blood pressure in hypertension; a controlled study. Circulation.

[12] Smithwick RH, Thompson JE (1953). Splanchnicectomy for essential hypertension; results in 1,266 cases. J Am Med Assoc.

[13] Bhatt DL, Bakris GL (2012). The promise of renal denervation. Cleve Clin J Med.

[14] Thukkani AK, Bhatt DL (2013). Renal denervation therapy for hypertension. Circulation.

[15] Myat A, Redwood SR, Qureshi AC (2013). Renal sympathetic denervation therapy for resistant hypertension: a contemporary synopsis and future implications. Circ Cardiovasc Interv.

[16] Krum H, Schlaich M, Whitbourn R (2009). Catheter-based renal sympathetic denervation for resistant hypertension: a multicentre safety and proof-of-principle cohort study. Lancet.

[17] Symplicity HTN-2 Investigators, Esler MD, Krum H, Sobotka PA, Schlaich MP, Schmieder RE, Böhm M (2010). Renal sympathetic denervation in patients with treatment-resistant hypertension (The Symplicity HTN-2 Trial): a randomised controlled trial. Lancet.

[18] Bhatt DL, Kandzari DE, O'Neill WW (2014). A controlled trial of renal denervation for resistant hypertension. N Engl J Med.

[19] Kandzari DE, Bhatt DL, Brar S (2015). Predictors of blood pressure response in the SYMPLICITY HTN-3 trial. Eur Heart J.

[20] Kandzari DE, Böhm M, Mahfoud F (2018). Effect of renal denervation on blood pressure in the presence of antihypertensive drugs: 6-month efficacy and safety results from the SPYRAL HTN-ON MED proof-of-concept randomised trial. Lancet.

[21] Townsend RR, Mahfoud F, Kandzari DE (2017). Catheter-based renal denervation in patients with uncontrolled hypertension in the absence of antihypertensive medications (SPYRAL HTN-OFF MED): a randomised, sham-controlled, proof-of-concept trial. Lancet.

[22] Azizi M, Schmieder RE, Mahfoud F (2018). Endovascular ultrasound renal denervation to treat hypertension (RADIANCE-HTN SOLO): a multicentre, international, single-blind, randomised, sham-controlled trial. Lancet.

[23] Azizi M, Schmieder RE, Mahfoud F (2019). Six-month results of treatment-blinded medication titration for hypertension control after randomization to endovascular ultrasound renal denervation or a sham procedure in the RADIANCE-HTN SOLO trial. Circulation.

[24] Böhm M, Kario K, Kandzari DE (2020). Efficacy of catheter-based renal denervation in the absence of antihypertensive medications (SPYRAL HTN-OFF MED Pivotal): a multicentre, randomised, sham-controlled trial. Lancet.

[25] Azizi M, Sanghvi K, Saxena M (2021). Ultrasound renal denervation for hypertension resistant to a triple medication pill (RADIANCE-HTN TRIO): a randomised, multicentre, single-blind, sham-controlled trial. Lancet.

[26] Moher D, Liberati A, Tetzlaff J, Altman DG (2009). Preferred reporting items for systematic reviews and meta-analyses: the PRISMA statement. J Clin Epidemiol.

[27] Sterne JA, Savović J, Page MJ (2019). RoB 2: a revised tool for assessing risk of bias in randomised trials. BMJ.

[28] Silverwatch J, Marti KE, Phan MT (2021). Renal denervation for uncontrolled and resistant hypertension: systematic review and network meta-analysis of randomized trials. J Clin Med.

[29] Bakris GL, Townsend RR, Liu M (2014). Impact of renal denervation on 24-hour ambulatory blood pressure: results from SYMPLICITY HTN-3. J Am Coll Cardiol.

[30] Azizi M, Sapoval M, Gosse P (2015). Optimum and stepped care standardised antihypertensive treatment with or without renal denervation for resistant hypertension (DENERHTN): a multicentre, open-label, randomised controlled trial. Lancet.

[31] Azizi M, Daemen J, Lobo MD (2020). 12-month results from the unblinded phase of the RADIANCE-HTN SOLO trial of ultrasound renal denervation. JACC Cardiovasc Interv.

[32] Azizi M, Mahfoud F, Weber MA (2022). Effects of renal denervation vs Sham in resistant hypertension after medication escalation: prespecified analysis at 6 months of the RADIANCE-HTN TRIO randomized clinical trial. JAMA Cardiol.

[33] Azizi M, Saxena M, Wang Y (2023). Endovascular ultrasound renal denervation to treat hypertension: the RADIANCE II randomized clinical trial. JAMA.

[34] Bakris GL, Townsend RR, Flack JM (2015). 12-month blood pressure results of catheter-based renal artery denervation for resistant hypertension: the SYMPLICITY HTN-3 trial. J Am Coll Cardiol.

[35] Bergland OU, Søraas CL, Larstorp AC (2021). The randomised Oslo study of renal denervation vs. antihypertensive drug adjustments: efficacy and safety through 7 years of follow-up. Blood Press.

[36] Warchol-Celinska E, Prejbisz A, Kadziela J (2018). Renal denervation in resistant hypertension and obstructive sleep apnea: randomized proof-of-concept phase II trial. Hypertension.

[37] Chen W, Ling Z, Du H (2016). The effect of two different renal denervation strategies on blood pressure in resistant hypertension: comparison of full-length versus proximal renal artery ablation. Catheter Cardiovasc Interv.

[38] Desch S, Okon T, Heinemann D (2015). Randomized sham-controlled trial of renal sympathetic denervation in mild resistant hypertension. Hypertension.

[39] de Jager RL, de Beus E, Beeftink MM (2017). Impact of medication adherence on the effect of renal denervation: the SYMPATHY trial. Hypertension.

[40] Fadl Elmula FE, Hoffmann P, Larstorp AC (2014). Adjusted drug treatment is superior to renal sympathetic denervation in patients with true treatment-resistant hypertension. Hypertension.

[41] Engholm M, Bertelsen JB, Mathiassen ON (2018). Effects of renal denervation on coronary flow reserve and forearm dilation capacity in patients with treatment-resistant hypertension. A randomized, double-blinded, sham-controlled clinical trial. Int J Cardiol.

[42] Fengler K, Rommel KP, Blazek S (2019). A three-arm randomized trial of different renal denervation devices and techniques in patients with resistant hypertension (RADIOSOUND-HTN). Circulation.

[43] Gao J-Q, Zhang H, Li L-Y, Wang X, Ye J, Liu Z-J (2021). Comparison of a 5 F microtube-irrigated ablation catheter and a general ablation catheter in the treatment of resistant hypertension with renal denervation. Cardiovasc Innov Appl.

[44] Gosse P, Cremer A, Pereira H (2017). Twenty-four-hour blood pressure monitoring to predict and assess impact of renal denervation: the DENERHTN study (renal denervation for hypertension). Hypertension.

[45] Hering D, Lambert EA, Marusic P (2013). Substantial reduction in single sympathetic nerve firing after renal denervation in patients with resistant hypertension. Hypertension.

[46] Jacobs L, Persu A, Huang QF (2017). Results of a randomized controlled pilot trial of intravascular renal denervation for management of treatment-resistant hypertension. Blood Press.

[47] Kario K, Ogawa H, Okumura K (2015). SYMPLICITY HTN-Japan - first randomized controlled trial of catheter-based renal denervation in Asian patients. Circ J.

[48] Kario K, Mahfoud F, Kandzari DE (2023). Long-term reduction in morning and nighttime blood pressure after renal denervation: 36-month results from SPYRAL HTN-ON MED trial. Hypertens Res.

[49] Kario K, Yokoi Y, Okamura K (2022). Catheter-based ultrasound renal denervation in patients with resistant hypertension: the randomized, controlled REQUIRE trial. Hypertens Res.

[50] Mahfoud F, Kandzari DE, Kario K (2022). Long-term efficacy and safety of renal denervation in the presence of antihypertensive drugs (SPYRAL HTN-ON MED): a randomised, sham-controlled trial. Lancet.

[51] Mathiassen ON, Vase H, Bech JN (2016). Renal denervation in treatment-resistant essential hypertension. A randomized, SHAM-controlled, double-blinded 24-h blood pressure-based trial. J Hypertens.

[52] Oliveras A, Armario P, Clarà A, Sans-Atxer L, Vázquez S, Pascual J, De la Sierra A (2016). Spironolactone versus sympathetic renal denervation to treat true resistant hypertension: results from the DENERVHTA study - a randomized controlled trial. J Hypertens.

[53] Pekarskiy SE, Baev AE, Mordovin VF (2017). Denervation of the distal renal arterial branches vs. conventional main renal artery treatment: a randomized controlled trial for treatment of resistant hypertension. J Hypertens.

[54] Peters CD, Mathiassen ON, Vase H (2017). The effect of renal denervation on arterial stiffness, central blood pressure and heart rate variability in treatment resistant essential hypertension: a substudy of a randomized sham-controlled double-blinded trial (the ReSET trial). Blood Press.

[55] Rosa J, Widimský P, Toušek P (2015). Randomized comparison of renal denervation versus intensified pharmacotherapy including spironolactone in true-resistant hypertension: six-month results from the Prague-15 study. Hypertension.

[56] Saxena M, Shour T, Shah M (2018). Attenuation of splanchnic autotransfusion following noninvasive ultrasound renal denervation: a novel marker of procedural success. J Am Heart Assoc.

[57] Schmieder RE, Ott C, Toennes SW (2018). Phase II randomized sham-controlled study of renal denervation for individuals with uncontrolled hypertension - WAVE IV. J Hypertens.

[58] Weber MA, Kirtane AJ, Weir MR (2020). The REDUCE HTN: REINFORCE: randomized, sham-controlled trial of bipolar radiofrequency renal denervation for the treatment of hypertension. JACC Cardiovasc Interv.

